# Extensive library of *Ruminococcus gnavus* phages revealing genomic and phenotypic diversity with novel clusters

**DOI:** 10.1128/spectrum.03672-25

**Published:** 2026-06-22

**Authors:** Arata Sakiyama, Ayaka Washizaki, Keiko Inaba-Hasegawa, Most Hushna Ara Naznin, Keigo Ide, Masayuki Takase, Yoriko Ibuki, Yuichi Sawaguchi, Shoichi Mitsunaka, Yutaro Ono, Islam Mohammad Shyful, Hiroki Ando

**Affiliations:** 1 Laboratory of Phage Biologics, Graduate School of Medicine, Gifu University 12785, Gifu, Japan; 2 R&DX, DigitalX, Astellas Pharma Inc., Ibaraki, Japan; 3 Venture Unit Engineered Phage Therapy, Discovery Accelerator, Astellas Pharma Inc, Ibaraki, Japan; 4 Center for One Medicine Innovative Translational Research (COMIT), Institute for Advanced Study, Gifu University 12785, Gifu, Japan; 5 Arrowsmith Inc., Ibaraki, Japan; Centre de Biologie Integrative, Toulouse, France

**Keywords:** *Ruminococcus gnavus*, bacteriophage, siphovirus morphology, temperate phage, intergenomic similarity

## Abstract

*Ruminococcus gnavus* is a prevalent member of human gut microbiota and is associated with inflammatory bowel disease. Phage-based microbiota engineering represents a promising approach for alleviating dysbiosis; however, knowledge regarding *R. gnavus* phages remains limited. We report the isolation and characterization of 29 novel *R. gnavus* phages from sewage samples. All isolates exhibited siphovirus morphology, with genome sizes ranging from 32.3 to 46.0 kbp, and represented 18 putative new genera and 24 new species. Whole-proteome clustering tools indicated that *R. gnavus* phages form a distinct new cluster and are divided into three clusters that could correspond to new subfamilies. Representative phages from each cluster were phenotypically characterized in detail, based on adsorption kinetics, one-step growth, and stress tolerance assays, which revealed diverse infection dynamics and structural features, such as unique globular appendages at distal tail fibers. Collectively, this study established the largest and most diverse *R. gnavus* phage library to date, providing fundamental insights into phage–host interactions and a valuable resource for the future development of phage-based strategies to modulate *R. gnavus* in the human gut.

**IMPORTANCE:**

*Ruminococcus gnavus* is a common gut commensal consistently associated with inflammatory bowel disease (IBD). Phage-based microbiota engineering has emerged as a promising strategy for alleviating dysbiosis. However, progress has been hampered by the limited availability of *R. gnavus* phages. In this study, we established and characterized a library of 29 novel *R. gnavus* phages. Whole-genome/proteome and phenotypic analyses revealed that these phages may represent a new taxonomic lineage and display variation in their infection strategies. This work substantially expands the known repertoire of *R. gnavus* phages and provides a critical resource for dissecting phage–host interactions in the human gut. Importantly, our findings lay the foundation for the future development of phage-based approaches to selectively modulate *R. gnavus* populations and inform therapeutic strategies for IBD.

## INTRODUCTION

*Ruminococcus gnavus* is a Gram-positive obligate anaerobic bacterium originally isolated from human feces in 1974 (1). It is now recognized as a prevalent member of human gut microbiota, detected in more than 90% of healthy adults and typically colonizing the gut during infancy (2–4). Certain *R. gnavus* strains, including ATCC 29149^T^ and ATCC 35913, possess strain-specific capabilities for degrading mucin and other host-derived glycans (5). These activities facilitate colonization of the mucosal niche and confer a competitive advantage. However, under inflammatory conditions, the same enzymatic activity may contribute to disruption of the gut ecosystem. Further, *R. gnavus* has been linked to inflammatory bowel disease (IBD), including Crohn’s disease and ulcerative colitis. Multiple metagenomic studies have reported significant enrichment of *R. gnavus* in the gut microbiota of patients with IBD compared with that in healthy controls (6–9). Moreover, *R. gnavus* induces inflammatory cytokine expression in mouse bone marrow-derived dendritic cells (6, 10). Collectively, these findings suggest that *R. gnavus* is involved in IBD-associated dysbiosis and may represent a potential therapeutic target.

Phages are viruses that specifically infect bacteria and are the most abundant biological entities on Earth (11). They are ubiquitous across diverse ecosystems, including soil, oceans, and the human body, and constitute a major component of the human gut virome (12). Phages have attracted renewed interest as therapeutic agents because of their bactericidal activity and high host specificity. Phage therapy is promising for combating multidrug-resistant bacteria that pose a serious threat to global health (13). Unlike broad-spectrum antibiotics, phages only kill their target bacteria and are thus increasingly considered as tools to modulate the gut microbiota to restore microbial balance in dysbiosis-related diseases (14). Targeting *R. gnavus* with phages in patients with IBD can therefore help alleviate disease symptoms.

Buttimer et al. were the first to report the isolation and genomic analysis of six *R. gnavus* phages from human feces and environmental samples (15). All six were temperate phages exhibiting siphovirus morphology and 36–38 kbp genomes, readily forming lysogens in the host. This study demonstrated the presence of *R. gnavus* phages in the gut and suggested the feasibility of phage-based control. However, these six phages showed limited genetic variation. Additionally, Liu et al. reported an *R. gnavus* phage, RG-TP1, via mitomycin C treatment during a broader survey of human gut phages (16). Although the genome sequence of RG-TP1 has been deposited, detailed genomic, phenotypic, and host range analyses have not been performed. Taken together, the currently available *R. gnavus* phages represent only a limited fraction of the potential genetic and biological diversity within this phage group. Thus, a larger and genetically diverse phage library, along with a deeper understanding of *R. gnavus* phage biology, particularly the host range and infection dynamics, is needed.

To this end, we isolated 29 phages growing on the well-known *R. gnavus* strain ATCC 35913 and characterized these phages from a genomic and taxonomic perspective, as well as in terms of their relationships with the hosts and their phenotypes. Overall, this library includes 18 new putative phage genera and 24 new putative phage species that deepen our understanding of *R. gnavus* phages and provide a foundation for microbiota editing using phage-based approaches.

## RESULTS

### Host selection for isolating *R. gnavus* phages

We obtained four *R. gnavus* strains as candidate hosts for phage isolation. All strains were originally isolated from human feces in diverse geographic locations, including Japan, Hawaii, the United Kingdom, and the United States (Table 1) (1, 17–19). Genome sequences were available for three strains (ATCC 29149^T^, ATCC 35913, and NBRC 114413), whereas JCM 31371 lacked genome information. To identify host-associated genomic features that might affect phage isolation, we examined prophage content and predicted antiphage defense systems in the sequenced genomes. All analyzed genomes harbored multiple prophage-related regions, including at least one intact prophage per strain (Table 1 and Table S1). Additionally, six to seven antiphage defense systems were identified in each genome (Table 1 and Table S2). Overall, the prophage content and predicted antiphage defense repertoires were broadly similar among the analyzed genomes.

**TABLE 1:** Characteristics of *R. gnavus* strains used in this study^*a*^

Strain	Isolation source	Country of isolation	Genome information	Mucin-degrading ability	Capsule status	Predicted prophage regions	Phage defense systems	References
ATCC 29149ᵀ	Healthy human feces	Hawaii, USA	Complete (NZ_CP027002)	+	+	1 intact, 3 questionable, 6 incomplete	6 types	(1, 10, 20, 21)
ATCC 35913	Healthy human feces	USA	Complete (ATCC Genome Portal)	+	–	2 intact, 1 questionable, 6 incomplete	7 types	(10, 17, 20, 21)
NBRC 114413	Healthy human feces	Japan	Complete (NZ_CP084014-5)	ND	ND	2 intact, 1 questionable, 11 incomplete	6 types	(19)
JCM 31371	Healthy human feces	United Kingdom	Not available	ND	ND	ND	ND	(18)

^*a*^ Information on *R. gnavus* strains used for phage isolation and host range analysis. Prophage regions were predicted using PHASTER and are reported as intact, questionable, or incomplete. Detailed results are provided in Table S1. Phage defense systems were predicted using PADLOC v2.0.0. Detailed results are provided in Table S2. The genome sequence of ATCC 35913 was obtained from the ATCC Genome Portal, as it was not publicly available in the NCBI database at the time of analysis. ND, not determined.

Mucin-degrading ability has been reported for the two ATCC strains but has not been characterized for NBRC 114413 (17, 20). The two ATCC strains harbor the intramolecular trans-sialidase gene *nanH*, which is part of a gene cluster associated with gut colonization (20–22), whereas NBRC 114413 lacks this gene cluster. Capsule production has been associated with both anti- and pro-inflammatory host responses, and strains harboring the capsule gene cluster have been isolated from both healthy donors and individuals with IBD (10, 20). Therefore, although the role of capsule production in IBD remains unclear, it may represent a contributing factor that warrants consideration. Among the strains examined in this study, ATCC 29149^T^ and ATCC 35913 carry a capsular polysaccharide gene cluster, whereas this cluster was not detected in NBRC 114413. Notably, ATCC 35913 does not produce a capsule due to mutations in this gene cluster (10).

Considering the potential application of phage therapy, we prioritized isolation hosts that resemble *R. gnavus* strains recovered from individuals with IBD. Based on these observations and previous studies, we selected the well-characterized strains ATCC 29149^T^ and ATCC 35913 for isolating *R. gnavus* phages, as they exhibit mucin-foraging activity and encompass both capsule-producing and non-producing phenotypes.

### Isolation of *R. gnavus* phages

We screened 155 samples, including 134 sewage samples from Gifu Prefecture, Aichi Prefecture, and Ibaraki Prefecture, Japan; two animal fecal samples from Astellas Pharma Inc., Japan; eight animal bedding samples; and 11 animal fecal samples from Gifu University, Japan, for phages targeting *R. gnavus* strains ATCC 29149^T^ or ATCC 35913. Twenty-nine phages infecting ATCC 35913 were recovered exclusively from sewage samples (Table 2). The morphology of plaques varied among phages, and 23 out of 29 phages formed clear plaques. The plaques formed by the remaining 6 phages were turbid (Table 2). No phages were isolated using ATCC 29149^T^ as the host strain, and none of the animal-derived samples yielded *R. gnavus* phages.

**TABLE 2:** General features of *R. gnavus* phages

Phage	Genome size (bp)	Cluster^a^	CDS	GC %	Integrase^b^	Repressor^c^	PCR detection^d^	Plaque morphology^e^	Isolation source	Accession no.
RgGU1	37,640	D	73	41.4	S	+	+	Clear	Sewage (Gifu)	LC889024
RgGU2	37,520	D	70	41.6	S	−	+	Turbid	Sewage (Aichi)	LC889035
RgGU3	33,669	C	60	42.5	S	−	+	Clear	Sewage (Aichi)	LC889046
RgGU4	32,325	D	64	42.1	S	−	+	Clear	Sewage (Aichi)	LC889047
RgGU5	33,478	D	67	42.7	S	−	+	Clear	Sewage (Aichi)	LC889048
RgGU6	33,504	C	71	42.6	S	+	+	Clear	Sewage (Aichi)	LC889049
RgGU7	36,347	C	58	42.3	S	−	+	Turbid	Sewage (Aichi)	LC889050
RgGU8	34,943	D	72	42.1	S	+	+	Clear	Sewage (Aichi)	LC889051
RgGU9	36,045	C	65	41.9	T	+	+	Clear	Sewage (Aichi)	LC889052
RgGU10	36,422	D	69	42.4	S	+	+	Clear	Sewage (Aichi)	LC889025
RgGU11	34,948	C	61	42.5	S	+	+	Turbid	Sewage (Aichi)	LC889026
RgGU12	37,927	C	72	42.4	S	+	+	Clear	Sewage (Aichi)	LC889027
RgGU13	34,741	D	63	42.2	S	−	+	Clear	Sewage (Aichi)	LC889028
RgGU14	33,622	D	69	42.2	–	−	−	Clear	Sewage (Aichi)	LC889029
RgGU15	37,407	D	67	42.3	S	+	+	Clear	Sewage (Aichi)	LC889030
RgGU16	32,818	D	63	42.7	T	−	−	Clear	Sewage (Aichi)	LC889031
RgGU17	38,175	D	73	41.9	S	+	+	Clear	Sewage (Gifu)	LC889032
RgGU18	37,492	D	71	42.3	S	+	+	Clear	Sewage (Gifu)	LC889033
RgGU19	46,025	B	83	41.8	S	+	+	Turbid	Sewage (Gifu)	LC889034
RgGU20	34,231	D	66	42.5	S	−	+	Clear	Sewage (Gifu)	LC889036
RgGU21	38,465	D	62	42.3	S	−	+	Turbid	Sewage (Gifu)	LC889037
RgGU22	34,943	D	72	42.1	S	+	+	Clear	Sewage (Gifu)	LC889038
RgGU23	38,261	D	72	42	S	+	+	Clear	Sewage (Gifu)	LC889039
RgGU24	37,481	D	73	42	S	+	+	Clear	Sewage (Gifu)	LC889040
RgGU25	35,630	D	68	42.1	S	+	+	Clear	Sewage (Ibaraki)	LC889041
RgGU26	32,888	D	63	42.2	S	+	+	Clear	Sewage (Ibaraki)	LC889042
RgGU27	36,983	D	70	42	S	+	+	Turbid	Sewage (Ibaraki)	LC889043
RgGU28	34,943	D	72	42.1	S	+	+	Clear	Sewage (Ibaraki)	LC889044
RgGU29	37,487	D	72	42	S	+	+	Clear	Sewage (Ibaraki)	LC889045

^a^ Cluster was defined using the VirClust analysis (Fig. 3B).

^b^ T: Integrase possessing a catalytic tyrosine residue (InterPro: IPR002104). S: Integrase possessing a serine-type DNA-binding recombinase domain (InterPro: IPR038109).

^c^ Putative repressor gene: transcriptional repressors with a Cro/CI-type helix–turn–helix domain (IPR001387).

^d^ Detection of phage genome from phage-resistant bacteria by PCR.

^e^ Morphology of plaques on ATCC 35913.

### Host specificity and killing activity of 29 phages against four *R. gnavus* strains

The infectivity of 29 phages was assessed by spot assays against the two strains used for the isolation step and two additional *R. gnavus* strains (Table 1). The use of four geographically distinct strains broadened the diversity of hosts examined. The efficiency of plating (EOP) measurements, normalized to ATCC 35913 (set as 1.0), and “lysis from without” (LO)—cell death due to massive simultaneous phage adsorption and/or phage-derived enzymes—were also recorded (23). Most phages showed plaques only on ATCC 35913, and none of them infected JCM 31371. Four phages showed infectivity across multiple tested *R. gnavus* strains: RgGU1 efficiently formed plaques on ATCC 29149^T^ (EOP = 1.1 ± 0.77); RgGU16 exhibited low EOP on ATCC 29149^T^ (EOP = 0.00088  ±  0.00098); RgGU19 formed plaques on *R. gnavus* ATCC 29149^T^ (EOP = 0.17 ± 0.072) and induced LO on NBRC 114413 at the undiluted titer (10^0^); and RgGU21 exhibited LO on both ATCC 29149^T^ (LO at 10^−2^) and NBRC 114413 (LO at 10^0^) (Table 3).

**TABLE 3:** Host range of *R. gnavus* phages^*a*^^,^^*b*^^,^^*c*^^,^^*d*^

Phage	*R. gnavus* strains		
	ATCC 29149^T^	JCM 31371	NBRC 114413
RgGU1	1.1 ± 0.77	–	–
RgGU2	–	–	–
RgGU3	LO (10^0^)	–	–
RgGU4	LO (10^0^)	–	–
RgGU5	LO (10^0^)	–	–
RgGU6	–	–	–
RgGU7	LO (10^−1^)	–	–
RgGU8	–	–	–
RgGU9	LO (10^0^)	–	–
RgGU10	–	–	–
RgGU11	–	–	–
RgGU12	–	–	–
RgGU13	LO (10^0^)	–	–
RgGU14	LO (10^0^)	–	–
RgGU15	LO (10^0^)	–	–
RgGU16	0.00088 ± 0.00098	–	–
RgGU17	LO (10^0^)	–	–
RgGU18	LO (10^0^)	–	–
RgGU19	0.17 ± 0.072	–	LO (10^0^)
RgGU20	–	–	–
RgGU21	LO (10^−2^)	–	LO (10^0^)
RgGU22	–	–	–
RgGU23	LO (10^0^)	–	–
RgGU24	LO (10^0^)	–	–
RgGU25	LO (10^0^)	–	–
RgGU26	LO (10^0^)	–	–
RgGU27	LO (10^0^)	–	–
RgGU28	–	–	–
RgGU29	LO (10^0^)	–	–

^*a*^ EOP (mean ± SD, *N* = 3): Efficiency of plating normalized to that on *R. gnavus* ATCC 35913 (set as 1.00).

^*b*^ LO (10^−n^): Lysis from without observed at above indicated dilution.

^*c*^ –, no lysis or no plaque formation observed.

^*d*^ All phages were spotted in 10-fold serial dilutions from 10^0^ to 10^−7^ at 1 × 10^8^ PFU mL^−1^ starting concentration.

**Fig 1:**
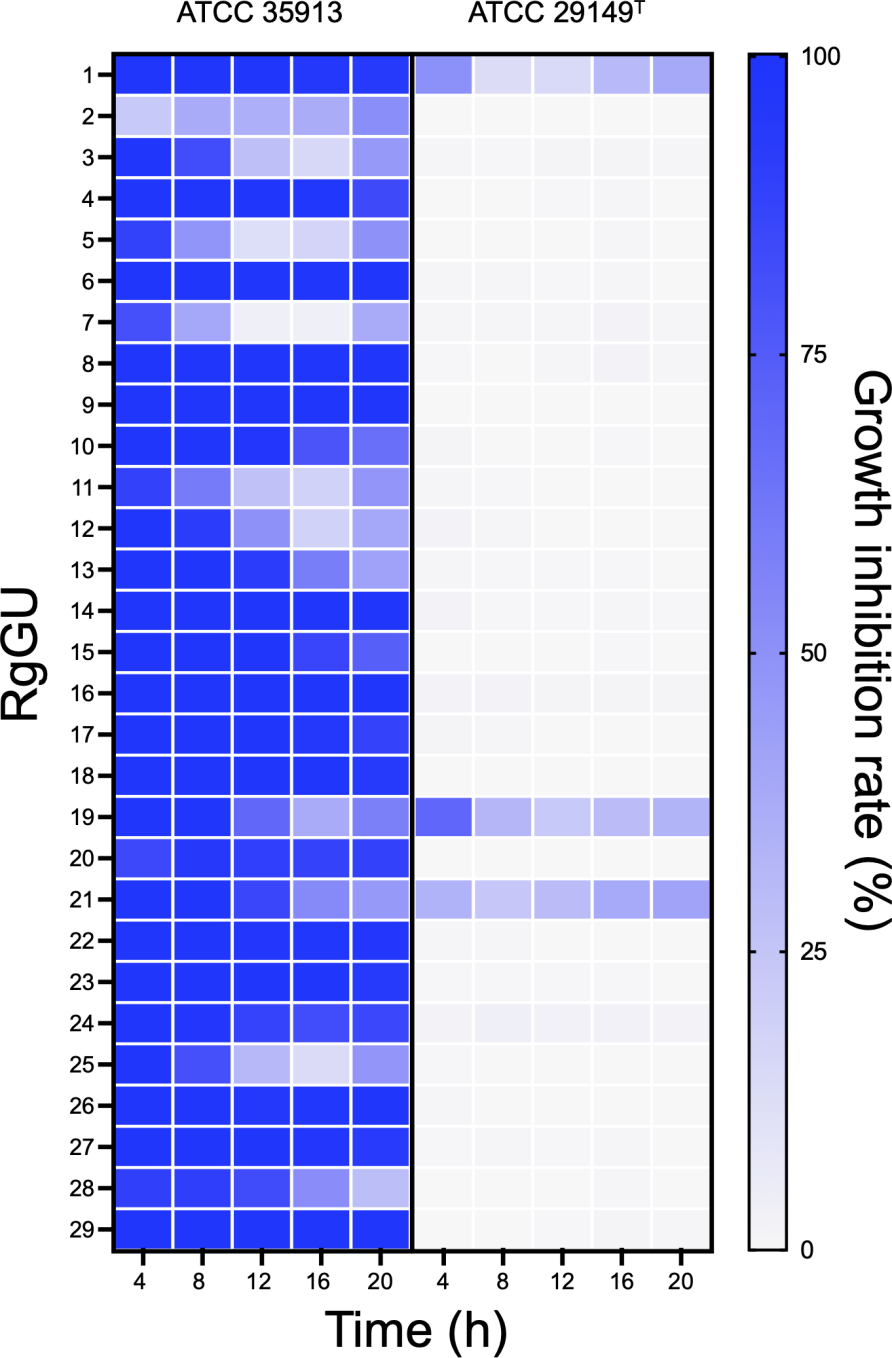
Heatmap representation of the killing activity of RgGU phages against *R. gnavus* ATCC 35913 and 29149^T^. OD_600_ values at 4, 8, 12, 16, and 20 hpi were extracted from the killing curves shown in Fig. S1, and the growth inhibition rate (%) at each time point was calculated as follows: (1 – OD_600 phage_ / OD_600 control_) × 100. Values higher than 100% and lower than 0% were set to 100% and 0%, respectively. Darker blue indicates a stronger suppression of bacterial growth. Rows represent individual phages, and columns represent post-infection time points. The results for ATCC 35913 and ATCC 29149^T^ are shown in the left and right five columns, respectively. Data represent the mean of three independent experiments.

Killing kinetics assays (multiplicity of infection [MOI] = 1) revealed rapid suppression of ATCC 35913 growth by most phages, resulting in 89%–100% growth inhibition, with the OD_600_ decreasing within 2.5 h post-infection (hpi), except for RgGU2, which showed delayed and incomplete lysis (Fig. 1 and Fig. S1). Sixteen phages (RgGU1, 4, 6, 8, 9, 14, 16–18, 20, 22–24, 26, 27, and 29) maintained >80% growth inhibition for up to 20 hpi, whereas the others allowed bacterial regrowth within 12 hpi. Early regrowth (4–8 hpi) with RgGU5, 7, and 11 suggested incomplete infection, rapid emergence of phage-resistant subpopulations, or lysogenization (Fig. 1 and Fig. S1).

At 8 hpi, bacterial growth was reduced to 48%, 38%, and 60% for RgGU5, 7, and 11, respectively (Fig. 1 and Fig. S2). On ATCC 29149^T^, measurable lytic activity was obtained only for RgGU1, 19, and 21. RgGU1 exhibited bacterial growth inhibition at 4 hpi (51%); however, inhibition decreased to 13% by 8 hpi, and regrowth was observed. RgGU19 showed 70% bacterial growth inhibition at 4 hpi, but this declined to 31% at 8 hpi, indicating regrowth. By contrast, RgGU21 showed 34% growth inhibition at 4 hpi and maintained sustained growth inhibition of approximately 24%–41% up to 20 hpi. No killing activity was observed on NBRC 114413 or JCM 31371 (Fig. S1).

### Nucleotide-level identity to registered phage genomes of 29 newly isolated *R. gnavus* phages

To assess the genomic relatedness of the 29 isolated *R. gnavus* phages to previously reported viruses, we conducted BLASTn searches against the NCBI Core_nt database, restricted to viral sequences (taxid:10239). For all isolates except RgGU4, the top BLASTn hits corresponded to partial viral genomes assembled from human gut metagenomic data sets (Table S3). Importantly, none of the newly isolated phages met the International Committee on Taxonomy of Viruses (ICTV) genus-level threshold (≥70% intergenomic identity) with any previously cultured phages. Overall, none of the newly isolated phages showed significant nucleotide identity with previously isolated phages. Additionally, BLASTn searches against bacterial genomes revealed that all 29 phages shared homologous regions with *Mediterraneibacter gnavus* (formerly *R. gnavus*) genomes (Table S4). Notably, homologous regions were identified in prophage regions of *R. gnavus* ATCC 29149 and NBRC 114413, whereas no closely related prophage sequences were detected in ATCC 35913.

### Genomic features of 29 *R. gnavus* phages

The genome sizes of the isolated phages ranged from 32,352 to 46,025 bp. The GC content was 41.4%–42.7%, and the predicted coding sequences (CDSs) numbered 58–83 (Table 2). No genes associated with antibiotic resistance or virulence were identified in the phage genomes. However, several phages encoded factors that may facilitate evasion of host restriction–modification (R–M) systems. Specifically, a Lar-like restriction alleviation protein (PF14354), known to counteract host restriction enzymes, was identified in RgGU1–4, 8, 10, 13, 15, and 17–29. Additionally, DNA methyltransferases (IPR001525) were detected in RgGU7, 9, 10, 12, 13, 18, 19, and 21. These proteins likely function as countermeasures against bacterial restriction enzymes that target foreign DNA, thereby enhancing phage survival during infection. In addition to R–M evasion systems, several phages encoded toxin–antitoxin (TA) modules. Notably, RgGU21 phage carried a complete HicAB module, whereas RgGU3 and 15 encoded only HicB homologs. The presence of a full HicAB system suggests a temperate lifestyle for the phage and a potential role of the system in addiction or abortive superinfection exclusion, whereby activation of the HicA toxin upon secondary infection may inhibit the propagation of competing phages. By contrast, phages encoding only HicB may represent degenerated TA systems or could function as regulatory or anti-defense factors, potentially interfering with host-encoded TA-mediated defense mechanisms (24). Interestingly, among the RgGU phages, only RgGU15 harbored a putative anti-CRISPR protein, indicating that this phage may possess a specific mechanism to evade host CRISPR–Cas defense systems.

To further investigate features related to phage life cycle and lysogeny, we examined genes associated with site-specific recombination and transcriptional regulation. Based on phage genome annotation, RgGU14 does not encode any potential integrase, whereas RgGU9 and RgGU16 encode a tyrosine-type integrase (InterPro: IPR002104) that mediates integration through a Holliday-junction intermediate and often requires accessory factors. By contrast, other phages possess an integrase with a serine-type DNA-binding recombinase domain (InterPro: IPR038109), which catalyzes concerted double-strand cleavage and strand exchange (25) (Table 2). The presence of these domains indicates that *R. gnavus* phages employ distinct recombination mechanisms, demonstrating their evolutionary diversity. Phages encoding an integrase are generally considered temperate because the enzyme mediates site-specific recombination between phages and host genomes. In addition to integrase, RgGU1, 6, 8–12, 15, 17–19, and 22–29 phages encoded genes annotated as transcriptional repressors with a Cro/CI-type helix–turn–helix domain (InterPro: IPR001387) (Table 2). This DNA-binding motif is a hallmark of phage repressors that bind to operator sequences for suppressing lytic gene expression, thereby maintaining lysogeny. By contrast, phages RgGU2–5, 7, 13, 14, 16, 20, and 21 lacked the putative repressor genes in this domain.

### Lysogenic potential in 29 *R. gnavus* phages

All seven phages reported to date have been described as temperate phages. To determine whether 29 phages exhibit similar lifestyles, we investigated their life cycles through functional assays. Phage lysates were spotted onto ATCC 35913 lawns, and phage-resistant colonies were isolated in three independent experiments. PCR analysis targeting phage-specific sequences in these colonies suggested lysogeny or pseudolysogeny for 27 phages (Table 2 and Fig. S2). For RgGU14 and RgGU16, 20 colonies were isolated and analyzed by colony PCR, but no PCR-positive colonies were detected (Table 2). Taken together with genomic features typically associated with temperate phages, these observations are consistent with the possibility of non-lytic states, including lysogeny or pseudolysogeny, in 27 phages, except for RgGU14 and RgGU16.

### Genome- and proteome-based classification of *R. gnavus* phages

**Fig 2:**
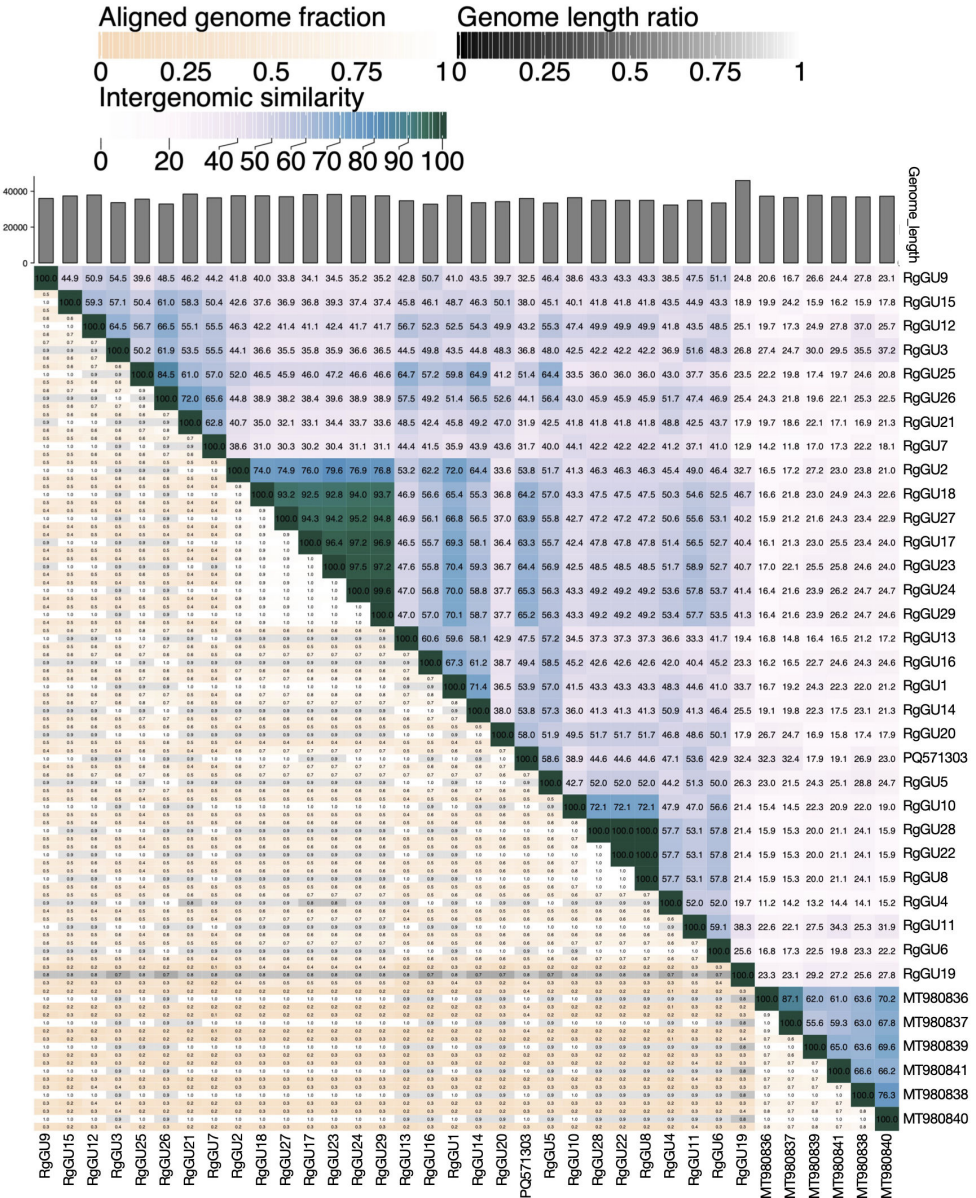
VIRIDIC-based intergenomic similarity analysis of 29 newly isolated and seven previously reported *R. gnavus* phages. Pairwise intergenomic nucleotide similarities were calculated using VIRIDIC v1.1 based on BLASTn-derived whole-genome alignments. The upper-right half of the matrix shows the percentage intergenomic similarity for each genome pair, color-coded from white (0%) to dark green (100%), as indicated by the similarity scale. The diagonal represents self-comparisons (100% intergenomic similarity). The lower-left half of the matrix displays three additional alignment parameters for each genome pair. At each intersection (row vs column), values are presented from top to bottom as follows: (**i**) aligned genome fraction 1, representing the proportion of the genome on the row that aligns to the genome on the column; (ii) genome length ratio between the two phages; and (iii) aligned genome fraction 2, representing the proportion of the genome on the column that aligns to the genome on the row. These parameters are color-coded according to their respective scales shown above the matrix. Values approaching 1.0 indicate nearly complete genome alignment and similar genome sizes, whereas lower values indicate partial genomic homology and/or genome length differences. Genome lengths (bp) are shown as bar plots along the right margin of the figure. Dashed lines indicate ICTV-recommended taxonomic thresholds (≥95% intergenomic similarity for species-level classification and ≥70% for genus-level classification). Newly isolated phages are indicated by the prefix “RgGU,” and previously reported phages (accession numbers: PQ571303 and MT980836–MT980841) are included for comparison.

Intergenomic similarity analysis using VIral Relatedness Inference by Distance Index Calculation (VIRIDIC) revealed that, according to the ICTV thresholds, the entire collection of 36 *R. gnavus* phages encompassed 23 putative genera (≥70% nucleotide intergenomic similarity) and 31 species (≥95% nucleotide intergenomic similarity) (Fig. 2). The six *R. gnavus* phages previously isolated by Buttimer et al. exhibited low intergenomic similarity to those isolated in this study, with the highest similarity (29.2%) observed between RgGU19 and phiRgPS_6 (MT980839) (Fig. 2). Among all pairwise comparisons, RG-TP1 (PQ571303) exhibited the highest intergenomic similarity (65.3%) with RgGU24. Among the 29 phages, intergenomic similarity analysis grouped them into 18 distinct genera. RgGU2, 17, 18, 23, 24, 27, and 29 formed the largest genus cluster, exhibiting intergenomic similarity ranging from 74.0% to 99.9%. Within this group, RgGU17, 23, 24, and 29 met the ICTV species-level threshold, suggesting that they represent the same viral species. Notably, RgGU24 and RgGU29 shared an exceptionally high similarity of 99.6%, despite being isolated from different sources—Gifu and Ibaraki, respectively. RgGU8, 22, and 28 belonged to the same species and were grouped in the same genus as RgGU10. RgGU8, 22, and 28 shared an almost identical nucleotide sequence (99.99% similarity; Fig. 2), despite being isolated from geographically distinct sites—Gifu, Aichi, and Ibaraki, respectively.

**Fig 3:**
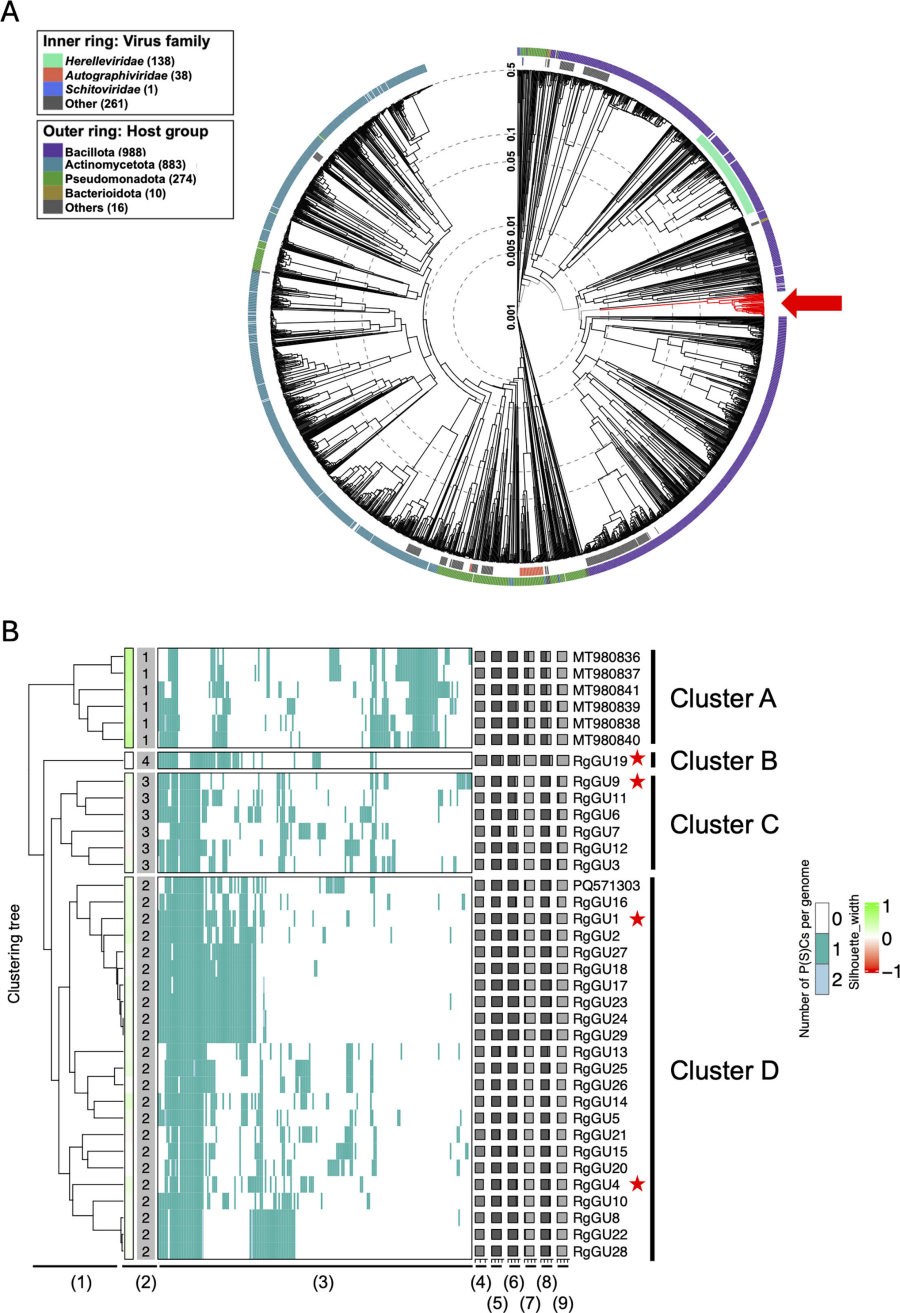
Whole-proteome–based relationships and VirClust hierarchical clustering of newly isolated and previously reported *R*. *gnavus* phages. (**A**) ViPTree-circular proteomic dendrogram of double-stranded DNA phage infecting prokaryotic hosts. The dendrogram was generated based on whole-proteome similarity using tBLASTx comparisons against the ViPTree reference database. This dendrogram includes 39 input phage genomes together with 2,260 reference dsDNA phage genomes that were identified as related phages under the default ViPTree settings. The outer ring indicates the host bacterial phylum of each phage. The inner ring shows the viral family classification assigned by ViPTree under default setting. The 29 phages isolated in this study, along with seven previously reported *R. gnavus* phages (accession numbers PQ571303 and MT980836–MT980841), are highlighted in red and indicated by red arrows to show their positions among related dsDNA phages. (**B**) Hierarchical clustering of *R. gnavus* phage genomes using VirClust. Labels (1)–(9) below Fig. 3B correspond to the following: (1) Intergenomic distances were calculated based on shared protein clusters using VirClust. (2) The relationships among phage genomes were visualized as a hierarchical dendrogram. Viral genome clusters (VGCs) were defined using a distance threshold of 0.625. Per the VirClust benchmarking framework, this threshold has been suggested to approximate subfamily-level groupings based on shared protein cluster similarity. Similarity within each VGC is indicated by color shading, with values approaching 1 shown in lime green (3). Distribution of viral genome protein clusters (PCs) is shown as a heatmap, where rows represent individual phage genomes and columns represent individual protein clusters. Color intensity reflects the number of proteins assigned to each cluster (4–9). Genome-specific statistics displayed to the right include: (4) genome length (bp); (5) fraction of shared proteins relative to total proteins; (6) fraction of proteins shared within a VGC; (7) fraction of proteins specific to a given VGC; (8) proportion of proteins shared outside the VGC; and (9) proportion of proteins shared exclusively outside the VGC. Phages marked with red stars were selected as representative isolates for subsequent genomic and phenotypic analyses.

To complement the nucleotide-based classification, proteomic relationships among the isolated *R. gnavus* phages were analyzed. Through proteome analysis using ViPTree, we compared the 29 newly isolated and seven previously reported *R. gnavus* phages (accession numbers: PQ571303, MT980836–MT980841) against a total of 2,260 related phage genomes. The analysis demonstrated that *R. gnavus* phages formed a single distinct lineage separated from other dsDNA phages (Fig. 3A). This finding supports the unique evolutionary position of *R. gnavus* phages within the broader virosphere and highlights their genomic distinctiveness. Following proteomic analysis using ViPTree, genome clustering analysis was performed using VirClust. Constituent viral genome clusters were delineated using a clustering distance threshold of 0.625, corresponding to the subfamily-level cutoff in VirClust (26). Application of this threshold resolved the phages into four distinct subfamily-level clusters, designated Cluster A–D in this study (Fig. 3B). Cluster A comprised six phages belonging to four genera that had previously been isolated by Buttimer et al. (15). In contrast, the newly isolated phages in this study were distributed among the remaining three clusters (Fig. 3B). Cluster B included RgGU19 as a single-member lineage. Cluster C comprised six phages representing six putative genera. The remaining 22 phages belonging to 12 putative genera, together with the previously reported phage RG-TP1, formed a separate lineage designated as Cluster D. To further investigate genome organization at the protein level, DiGAlign was employed to analyze protein-level genome alignment, first within individual clusters and subsequently across clusters, to assess conserved gene content and synteny. DiGAlign analysis of Cluster A revealed extensive conservation of structural and replication-associated genes at the protein level (Fig. S3A). Core virion structural modules, including terminase (*terS*/*terL*), major capsid protein (*mcp*), major tail protein (*mtp*), tape measure protein (*tmp*), and other tail-associated structural proteins, were highly conserved in both sequence identity and genomic organization. Genes involved in lysogeny, such as integrase (*int*) and repressor (*rep*), were also conserved within Cluster A. By contrast, genes associated with DNA replication, including DNA polymerase (*pol*), were not consistently conserved across cluster members. Furthermore, gene order and transcriptional orientation were largely maintained, indicating strong intra-cluster synteny. Variability was primarily observed in auxiliary or hypothetical genes located near the genome right end, suggesting that Cluster A phages share a cohesive and conserved genomic architecture (Fig. S3A). By contrast, Clusters C and D shared conserved virion structural genes, including terminase, capsid, and tail-associated proteins (Fig. S3B and S3C). However, genes related to lysogeny, such as *int* and *rep* genes, were not shared between these clusters. Nevertheless, similar to Cluster A, gene order and transcriptional orientation were largely conserved within each cluster, indicating strong synteny.

**Fig 4:**
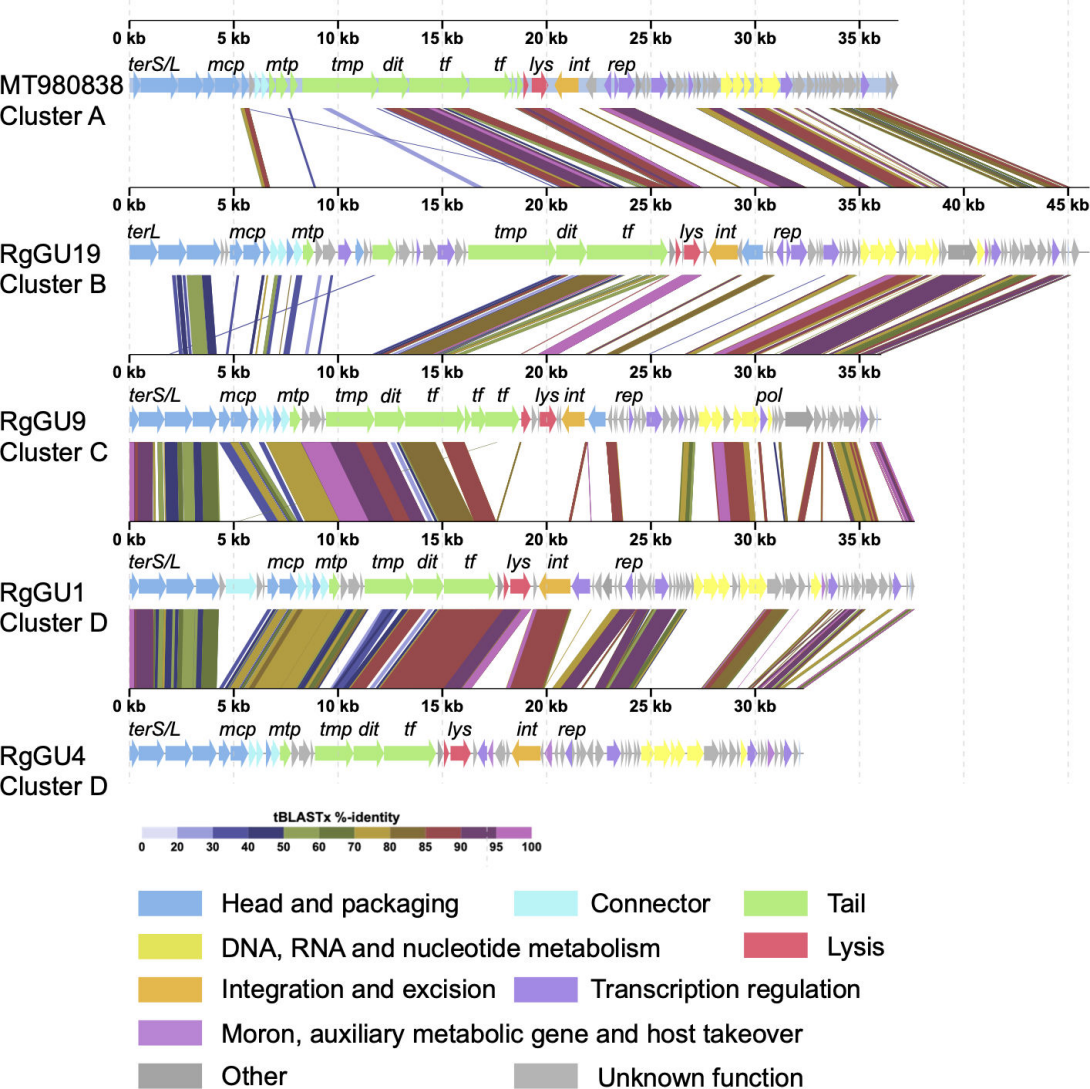
Protein-level genome alignment of representative *R. gnavus* phages from each VirClust-defined cluster (Clusters A–D), performed using DiGAlign. Conserved gene blocks are indicated by darker, continuous regions, reflecting a high intra-cluster similarity. Each gene is colored according to its predicted functional category based on the genome annotation. Abbreviated gene annotations are shown above selected coding sequences (CDSs), including *terS* (terminase small subunit), *terL* (terminase large subunit), *mcp* (major capsid protein), *mtp* (major tail protein), *tmp* (tape measure protein), *dit* (distal tail protein), *tf* (tail fiber protein), *lys* (endolysin), *int* (integrase), *rep* (repressor), and *pol* (DNA polymerase). Colors represent functional categories as follows: head and packaging, light blue; DNA, RNA, and nucleotide metabolism, yellow; connector, light cyan; tail, light green; lysis, red; integration and excision, orange; moron, auxiliary metabolic gene, and host takeover, pastel violet; transcription regulation, lavender purple; other, gray; and unknown function, light gray.

To perform inter-cluster comparisons, representative phages were selected from each cluster. MT980838 was chosen from Cluster A, RgGU19 from Cluster B, and RgGU9 from Cluster C. As Cluster D comprised multiple phages, two representatives, RgGU1 and RgGU4, were selected. RgGU1, 4, and 9 were chosen based on their ability to form clear plaques on ATCC 35913 and their lytic activity against ATCC 29149^T^. Inter-cluster comparison revealed that only the tail fiber and distal tail protein genes were shared across Clusters A–D, whereas other genes showed limited conservation. By contrast, Clusters B and C shared virion structural and genome packaging–related genes, such as those encoding capsid, tail proteins, and terminase (Fig. 4). This pattern suggests that tail-associated structural modules are highly conserved across lineages, likely reflecting functional constraints related to host recognition. By contrast, other genomic regions appear to have diversified substantially, consistent with modular evolution. Despite this diversification, overall gene organization and transcriptional orientation remained broadly similar across all clusters. Subsequently, these representative phages were subjected to detailed phenotypic and genomic characterization to further examine their biological properties and infection dynamics.

### Morphology of representative *R. gnavus* phages

**Fig 5:**
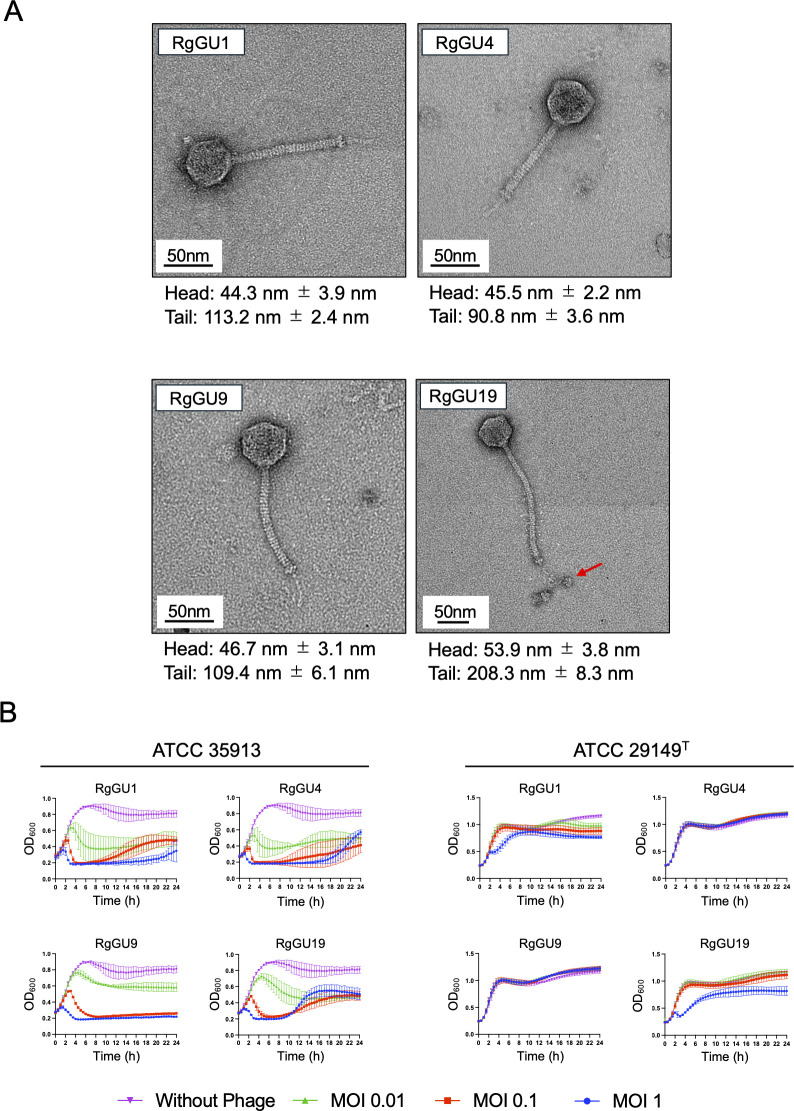
Morphological and growth characterization of representative *R*. *gnavus* phages. (**A**) Phage lysates were negatively stained with uranyl acetate and visualized using a TEM JEM-2100 (JEOL). Scale bar: 50 nm. The lengths of the head and tail were measured using ImageJ (NIH, USA; version 1.54g). The mean values and standard deviations of at least seven phage particles are shown. Red arrow showed appendages observed at the distal part of tail fibers. (**B**) Killing curves of *R. gnavus* strains ATCC 35913 and ATCC 29149^T^ were determined as follows: host bacteria were infected with representative *R. gnavus* phages at MOIs ranging from 0.01 to 1. Bacterial growth without the phage was used as a control. All experiments were performed in triplicate, and the error bars represent the mean ± SD.

Transmission electron microscopy (TEM) confirmed that all representative phages have siphovirus morphology with long, flexible tails. The head diameters were similar (39–62 nm), but tail lengths varied: the tails of RgGU1 and RgGU9 measured 99–117 nm; RgGU4 had shorter tails (90.8 nm ± 3.6 nm); and RgGU19 had the longest tail (208.3 nm ± 8.3 nm). Appendages at the distal tail fibers were observed in 89.2% of RgGU19 particles (Fig. 5A).

### Stability under pH and temperature stress

All tested phages remained stable up to 50°C. RgGU4 and RgGU19 showed reduced titers by more than 100-fold at 60°C; however, RgGU1 and RgGU9 retained infectivity, indicating relative heat tolerance (Fig. S4A). None of the strains showed loss of infectivity at pH 4−10. RgGU4 and RgGU19 remained infectious at pH 11, although a 100-fold reduction in titer was observed for RgGU19 (Fig. S4B). This result suggests that RgGU4 has greater stability over a broad pH range than the other tested phages.

### Killing kinetics of representative phages on ATCC 35913 and ATCC 29149^T^

To precisely analyze the killing kinetics of *R. gnavus* phages, ATCC 35913 cells were infected with phages at MOI = 0.01, 0.1, or 1, and the OD_600_ was measured every 30 min. All phages suppressed bacterial growth at an MOI of 1 after 2.5 h of infection. The increase in OD_600_ due to bacterial growth was suppressed in an MOI-dependent manner (Fig. 5B). Notably, RgGU9 maintained the suppression of bacterial growth at an MOI of 0.1 for 24 hpi, whereas RgGU19 showed regrowth within 12 hpi (Fig. 5B). On ATCC 29149^T^, growth suppression was observed for RgGU1 at approximately 2.5 hpi at an MOI of 1, whereas at MOIs of 0.1 and 0.01, growth suppression became apparent at around 10 hpi. RgGU19 showed growth inhibition only at an MOI of 1. Phages RgGU4 and RgGU9, which did not form plaques on solid media, were also unable to reduce bacteria growth under liquid conditions (Fig. 5B).

### Adsorption kinetics and one-step growth of *R. gnavus* phages

**Fig 6:**
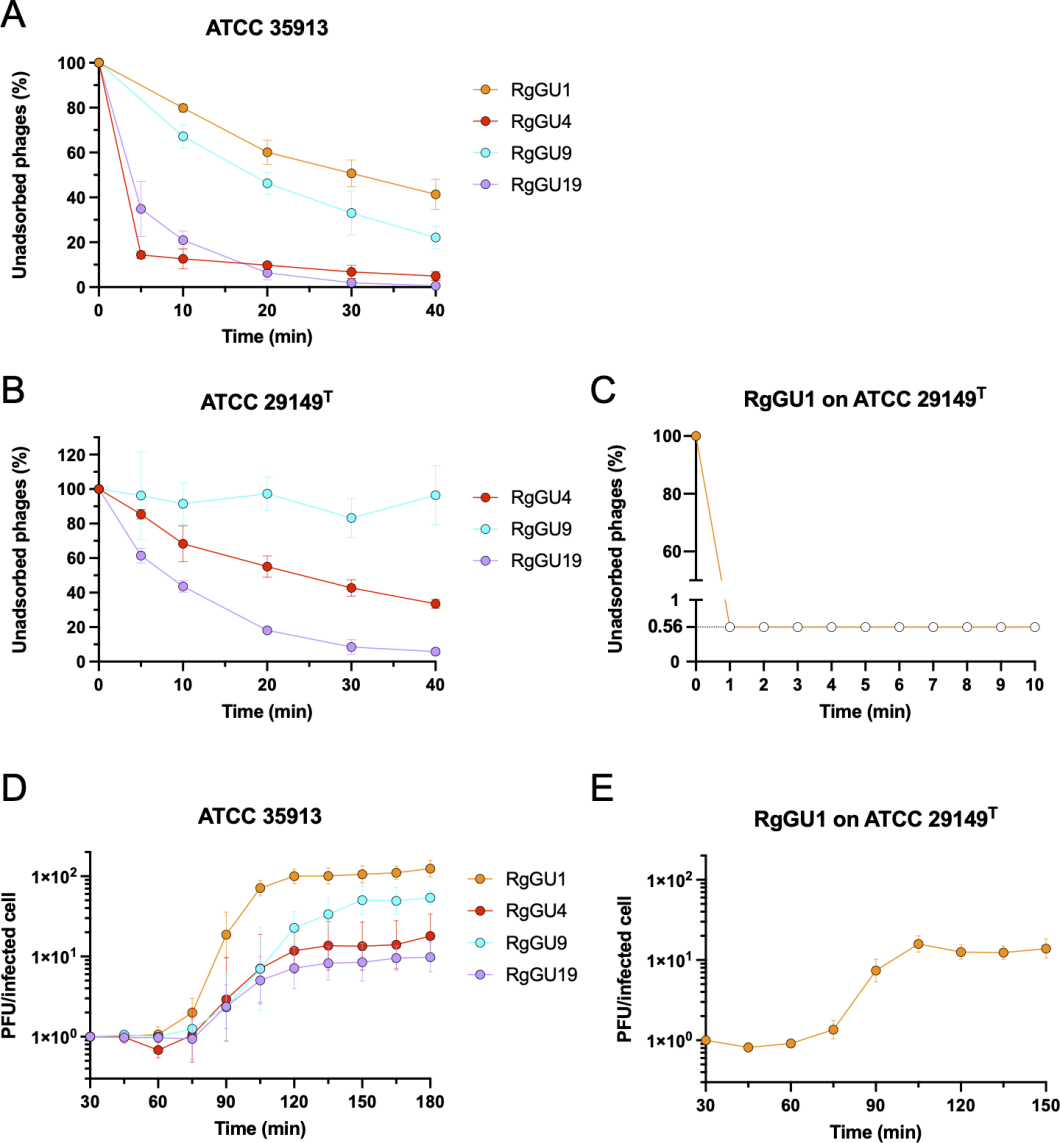
Adsorption kinetics and one-step growth curve of representative *R. gnavus* phages. (**A**) Adsorption kinetics of RgGU1, 4, 9, and 19 by *R. gnavus* ATCC 35913. Host bacteria were infected with each phage and incubated at 37°C. The titer of the unadsorbed phages was determined at the indicated time points. Experiments were performed in triplicate, and the mean ± SD is shown. (**B**) Adsorption kinetics of RgGU4, 9, and 19 on *R. gnavus* ATCC 29149ᵀ. (**C**) Adsorption kinetics of RgGU1 on *R. gnavus* ATCC 29149ᵀ. The assay was performed as described in panel A. The detection limit (shown as a dotted line) was 0.56%, and the open circles indicate values below this threshold. (**D**) One-step growth curves of RgGU1, 4, 9, 19 in *R. gnavus* ATCC 35913. Host bacteria were infected at an MOI of 0.1 and incubated at 37°C. The titer of the progeny phages was determined at the indicated time points. All experiments were performed in triplicate and independently repeated three times. Data are presented as mean ± SD. (**E**) One-step growth curve of RgGU1 in *R. gnavus* ATCC 29149^T^. The assay was performed as described in panel C.

To elucidate the growth kinetics of *R. gnavus* phages, we examined their adsorption kinetics and one-step growth curves. Adsorption assays revealed rapid binding of RgGU4 and RgGU19 to ATCC 35913 (∼80% adsorbed within 10 min), whereas RgGU1 adsorbed more slowly (∼50% after 30 min) (Fig. 6A). Interestingly, RgGU1 was adsorbed almost instantaneously onto ATCC 29149^T^ (<1% unbound at 1 min) (Fig. 6B). One-step growth curves showed that RgGU1 had the shortest latent period (burst starting at 75 min), whereas the other phages released progeny at 90 min (Fig. 6C). Burst sizes varied widely: RgGU1, 9, and 4 yielded 108.2, 53.6, and 20.1 PFU/cell, respectively. When infecting ATCC 29149^T^, the latent period of RgGU1 was unchanged, but the burst size dropped to 22.3 PFU per cell (Fig. 6D), likely contributing to its turbid plaque phenotype on this host.

## DISCUSSION

*R. gnavus* is an important constituent of human gut microbiota and is associated with IBD (6–9), making it a promising target for microbiota modulation to alleviate disease symptoms. Phage therapy represents a potential therapeutic strategy; however, only six *R. gnavus* phages have been characterized to date (15). Therefore, expanding the available phage library and performing detailed characterizations constitute critical steps. In this study, we isolated 29 novel phages from sewage samples and characterized their genomes and biological properties. Further characterization of four representative isolates revealed additional functional and structural variations in the phage library.

All isolated phages were recovered exclusively using *R. gnavus* ATCC 35913 as the host, whereas no phage was obtained with ATCC 29149ᵀ. Previous studies have shown that certain *R. gnavus* strains possess capsules and harbor candidate gene clusters for capsular polysaccharide biosynthesis (10). ATCC 35913 lacks a capsule because of multiple nonsense mutations in the cluster (10). Bacterial capsules are known to hinder phage adsorption by masking cell surface receptors, as reported for *Staphylococcus simulans*, *S. aureus*, and *Escherichia coli* (27–29). Thus, the presence of a capsule in ATCC 29149ᵀ likely explains the difficulty in isolating phages using this strain, whereas the absence of a capsule in ATCC 35913 may have facilitated phage isolation. Furthermore, ATCC 29149^T^ and NBRC 114413 were isolated from an individual of Japanese descent and from Japan, respectively. This raises the possibility that shared environmental phage populations may have influenced the prophage composition of these strains. Indeed, the 29 newly isolated phages share partial genomic identity with prophage regions present in the genomes of ATCC 29149^T^ and NBRC 114413. Such pre-existing prophage elements could potentially confer superinfection immunity or impose genetic compatibility constraints. Consequently, these factors may have influenced the relative difficulty in isolating phages from Japanese sewage using ATCC 29149^T^ as a host.

Additionally, we screened not only sewage samples but also animal fecal and bedding samples for phage isolation; however, no phages were recovered from these animal-derived sources. Recent metagenomic analyses have demonstrated that *R. gnavus* exhibits host-associated phylogenetic divergence between humans and companion animals such as cats and dogs (30). The absence of phage isolation from animal-derived samples in this study may partly reflect the use of human-derived *R. gnavus* strains (ATCC 29149^T^ and ATCC 35913) as isolation hosts. Although the lack of phage recovery cannot be attributed solely to host specificity, host-associated lineage divergence represents a plausible contributing factor.

The host range analysis conducted in this study revealed that *R. gnavus* phages generally exhibit relatively narrow infectivity profiles. While many phages showed high infectivity toward the isolation host ATCC 35913, their EOP varied substantially across other *R. gnavus* strains. These findings suggest that, even within the same bacterial species, host receptor structures, surface polysaccharides, R–M systems, and pre-existing prophage-mediated superinfection immunity strongly influence infection outcomes. Notably, in ATCC 29149ᵀ, most phages displayed markedly reduced EOP, only LO, or completely lost infectivity. This strain harbors prophage regions partially homologous to the newly isolated phages, and superinfection exclusion may therefore contribute substantially to the reduced infectivity observed. By contrast, RgGU1 and RgGU19 exhibited EOP values of 1.1 ± 0.77 and 0.17 ± 0.072, respectively. Despite lacking any known superinfection evasion factors, these phages retained infectivity, suggesting that unidentified molecular mechanisms may enable them to overcome prophage-derived defenses. Furthermore, the majority of isolated phages showed little to no infectivity against NBRC 114413 and JCM 31371 strains, with only RgGU19 and RgGU21 producing LO on the NBRC strain. Collectively, these results indicate that heterogeneity in cell surface architecture and antiphage defense repertoires among *R. gnavus* lineages plays a major role in determining phage susceptibility. However, several limitations should be acknowledged. The number of strains included in host range analysis was limited, complete genomic information was unavailable for the JCM strain, and only isolates derived from healthy individuals were examined. Therefore, broader investigations incorporating more diverse clinical isolates will be required to fully define host specificity within *R. gnavus*.

VIRIDIC-based intergenomic identity analysis revealed high genomic diversity among the isolates. Together with the seven previously described *R. gnavus* phages, the data set encompassed 23 putative genera and 31 species. Previously, the six *R. gnavus* phages isolated by Buttimer et al. were classified into four genera. By contrast, the newly isolated phages in this study were distributed across 18 putative genera, demonstrating broad genomic diversity. Furthermore, in VirClust analysis, these phages were grouped into four subfamily-level clusters, suggesting divergence within the *R. gnavus* phage population. Notably, the phages isolated in this study formed clusters distinct from those reported by Buttimer et al., indicating that they are distantly related. The broad taxonomic diversity observed, encompassing 18 putative genera, may in part reflect the use of sewage samples collected over a 2-year period from geographically distant sites (Gifu, Aichi, and Ibaraki Prefectures), which likely contributed to this outcome. Phage isolation efforts from diverse geographical regions may contribute to increasing the worldwide diversity of phage libraries.

BLASTn analysis revealed that all 29 isolated phages share homologous genomic regions with *R. gnavus* sequences detected in human gut metagenomic data sets, as well as with genomes of cultured *R. gnavus* isolates. The detection of homologous sequences within bacterial genomes supports the possibility that these phages may exhibit temperate lifestyles or are derived from temperate phages. In this study, phage-resistant bacterial colonies were isolated for each of the 29 phage isolates. To avoid detecting phages merely adsorbed to or remaining outside bacterial cells, three rounds of single-colony isolation were performed, followed by colony PCR to confirm the presence of phage genomes. Phage genomes were detected in 27 of the 29 isolates. These results suggest that the phages isolated in this study likely adopt a temperate lifestyle, similar to previously reported *R. gnavus* phages. However, as the integration sites were not identified, it was not possible to distinguish between true lysogeny and pseudolysogeny. According to genomic analyses, although both RgGU9 and RgGU16 encode tyrosine-type integrases, phage genome was detected only in RgGU9-resistant bacteria and not in RgGU16-resistant bacteria. Tyrosine-type integrases typically require a repressor gene to suppress lytic activation and thereby maintain lysogeny (31). The absence of an identifiable repressor in RgGU16, together with the failure to detect phage genomes in RgGU16-resistant bacteria, suggests that stable lysogeny or pseudolysogeny may not have been established, and RgGU16 could correspond to an ex-temperate phage. By contrast, other potentially temperate phages encoded serine-type integrases, suggesting the possibility of lysogeny. If the other phages in the library encoded serine-type integrases, any known repressors could also be identified on RgGU2–5, 13, 20, and 21. However, these phages did not encode any known repressors, which are typically required for maintaining lysogeny. Notably, some temperate phages harboring serine-type integrases—such as *Bacillus subtilis* phage SPβ and mycobacteriophage BPs—possess atypical repressors that lack a Cro/CI-type helix–turn–helix domain (32, 33). This raises the possibility that our phages lacking Cro/CI-type repressors may similarly employ uncharacterized regulatory proteins or atypical mechanisms to control the lysogeny–lysis switch. Consistently, RgGU2 and RgGU7 carry a gene annotated as a transcriptional repressor, which may represent a candidate regulator of such an alternative pathway. However, experimental validation is required to determine whether these regulators indeed mediate the lysogenic–lytic decision.

To gain deeper insights into *R. gnavus* phage biology, four isolates representing subfamily-level clusters and phenotypic groups (RgGU1, 4, 9, and 19) were characterized in detail. Phenotypic analyses revealed striking variations among the representative phages. RgGU1, 4, and 9 formed clear plaques, whereas RgGU19 formed turbid plaques on ATCC 35913. Temperate phages are generally thought to form turbid plaques, while lytic phages tend to form clear plaques (34). However, among the 29 phages isolated in this study, no correlation was observed between plaque morphology and putative lysogenicity. Recent reports have demonstrated that even the same phage can form either clear or turbid plaques depending on experimental conditions (35). Additionally, *R. gnavus* phages isolated in previous studies exhibited clear plaque morphology despite being temperate (15). Therefore, under our experimental conditions, plaque morphology is unlikely to be determined solely by the phage life cycle but rather influenced by other parameters, such as adsorption rate, burst size, and lysogenization efficiency. Among the three phages that formed clear plaques, RgGU1 showed relatively slow adsorption kinetics but a large burst size (>100 PFU/cell), whereas RgGU4 exhibited rapid adsorption but only a moderate burst size (20.1 PFU/cell). RgGU9 demonstrated moderate adsorption with a relatively large burst size. These characteristics may explain their ability to produce clear plaques. Additionally, beyond these factors, the lytic features may be explained by the presence of pre-existing prophages (active or remnant) at the integration site in the host genome, which could prevent successful lysogenization. Notably, the host strain used in this study is known to harbor prophage regions within its genome, supporting this possibility. Although we could not determine the lysogenization rate of each phage in this study, such information would likely allow a more precise explanation of plaque morphology. Notably, RgGU1 was adsorbed almost instantaneously onto ATCC 29149^T^, with the amount of unadsorbed phage dropping below the detection limit within 1 min. Because ATCC 29149^T^ produces a diffuse capsule that extends over a wide meshwork (10), RgGU1 may have been trapped and inactivated by the capsule material before stable adsorption, potentially leading to an overestimation of adsorption rates. Similarly, progeny phages might be sequestered by the capsule after release, preventing the infection of new cells and leading to an apparent reduction in burst size.

TEM confirmed that all four phages have siphovirus morphology. Notably, RgGU19 possessed globular appendages at the distal ends of their tail fibers. To our knowledge, similar globular structures have only been described in the *Bordetella* phage BPP-1 with bilobed globular tail fiber termini (36). Although BPP-1 has podovirus morphology, it represents the only available precedent for such globular tail fiber structures. In phages with siphovirus morphology, the distal structures typically include elongated tail fibers or tail tips (37, 38), but no globular appendages. Notably, the spherical appendages in RgGU19 were repeatedly observed at the distal end of the tail, suggesting that they may represent a structured component rather than incidental contaminants. However, whether the appendages observed in RgGU19 are bona fide phage structural proteins or bacterial contaminants adhering to tail fibers remains unclear. Nevertheless, this observation highlights the structural novelty of *R. gnavus* phages and warrants further proteomic and functional studies.

We established a library of 29 *R. gnavus* phages classified into 18 putative genera spanning three subfamily-level lineages, of which 27 exhibited features consistent with temperate or ex-temperate lifestyle. Because temperate phages can integrate into host bacterial genomes rather than consistently lyse them (39, 40), their utility for microbiota modulation may be limited. In addition, superinfection immunity and the potential for horizontal gene transfer raise concerns regarding their therapeutic application (41, 42). Consequently, genetic engineering is required when adapting temperate phages for therapeutic use. Recent advances in phage engineering, including CRISPR-Cas-based editing, recombineering, and synthetic engineering, provide promising avenues for overcoming these limitations (43–45). Although genetic manipulation of *R. gnavus* phages has not yet been reported, our diverse phage library provides a valuable resource for selecting candidate parental phages.

This study also has several limitations. The two ATCC strains used as isolation hosts are widely employed as model strains for investigating *R. gnavus*-associated diseases; however, both were originally isolated from healthy individuals. Additionally, host range analyses were performed using a limited number of strains and therefore may not fully represent the intraspecies diversity of *R. gnavus*. Future studies incorporating a broader range of disease-associated isolates will help clarify the host specificity and therapeutic potential of these phages within the gut environment.

Overall, our findings expand the currently available phage resources targeting *R. gnavus* and provide an important foundation for the development of phage-based strategies aimed at the targeted modulation of *R. gnavus* within the human gut microbiota.

## MATERIAL AND METHODS

### Bacterial strains and culture conditions

*R. gnavus* ATCC 29149^T^ and ATCC 35913 were purchased from the American Type Culture Collection (ATCC), whereas JCM 31371 and NBRC 114413 were obtained from the Japan Collection of Microorganisms (JCM) and the Biological Resource Center, National Institute of Technology and Evaluation (NBRC), respectively. All *R. gnavus* strains were cultured on modified GAM agar (Shimadzu, Japan) supplemented with 2 mM CaCl_2_, 0.95% (wt/vol) glucose, and 0.27% (wt/vol) sodium acetate (mGAM-CGA agar), or in modified GAM broth (Shimadzu, Japan) supplemented with 2 mM CaCl_2_, 0.95% (wt/vol) glucose, and 0.27% (wt/vol) sodium acetate (mGAM-CGA broth). All manipulations were carried out at 37°C in an anaerobic chamber (5% H_2_, 5% CO_2_, 90% N_2_) (BACTRON300, Sheldon Manufacturing Inc, USA).

### Genomic analysis of prophage content and antiphage defense systems

For the analysis of prophage content and antiphage defense systems in the host strains, the complete genome sequences of *R. gnavus* ATCC 29149^T^ (NZ_CP027002) and NBRC 114413 (NZ_CP084014-NZ_CP084015) were retrieved from the National Center of Biotechnology Information (NCBI). As the sequence of ATCC 35913 is not available in the NCBI database, it was obtained from the ATCC Genome Portal (https://genomes.atcc.org/genomes/3e9737a2f00b44b0). Prophage regions in the host genomes were identified using PHASTER (PHAge Search Tool Enhanced Release) (https://phaster.ca) (46), accessed in February 2026. Antiphage defense systems were predicted using PADLOC v2.0.0 under default settings. System classification and protein annotations were assigned based on HMM matches against the PadlocDB v2.0.0 database (47).

### Environmental sample collection and phage screening

Environmental samples including sewage, animal feces, and animal bedding were collected for phage isolation against *R. gnavus*. Sewage samples were centrifuged at 5,000 × *g* for 10 min at room temperature (RT), and the resulting supernatants were filtered through 0.22 µm pore-size filters (Sartolab RF500 PES, Sartorius, Germany). Animal feces and bedding samples were mixed with an equal volume (wt/vol) of phage buffer (1 mM CaCl_2_, 10 mM MgSO_4_, 68 mM NaCl, and 10 mM Tris-HCl [pH 7.5]) or PBS (−) (Wako, Japan), thoroughly vortexed, and centrifuged at 10,000 × *g* for 15 min at 4°C. The supernatants were sequentially filtered through 1.2 µm (Minisart, CA, Sartorius, Germany), 0.45 µm (Millex-HV, PVDF, Millipore, USA), and 0.22 µm (Millex-HV, PES, Millipore, USA) pore-size filters.

The resulting filtrates were then used for phage enrichment. Each filtrate was mixed with an equal volume of double-strength mGAM-CGA broth, subsequently inoculated with a small aliquot from a liquid culture of the isolation host *R. gnavus* (ATCC 29149^T^ or ATCC 35913), followed by anaerobic incubation at 37°C overnight. The enriched cultures were centrifuged at 5,000 × *g* for 5 min at RT, and the supernatants were collected. For phage detection, the enriched supernatant, an overnight culture of *R. gnavus*, and 3 mL of 0.4% mGAM-CGA broth soft agar were mixed and overlaid onto mGAM-CGA agar plates. After anaerobic incubation at 37°C overnight, the plaques were isolated. Phages were purified via two additional rounds of infection and single-plaque isolation. High-titer phage lysates were prepared from a single isolated phage suspension for subsequent analyses.

### Preparation of phage lysates

For phage lysate preparation, phages at 1 × 10^4^–1 × 10^5^ PFU were plated onto a lawn of ATCC 35913 and incubated at 37°C overnight. Once sufficient lysis was observed, 7 mL of phage buffer was added to the plate and incubated at RT for 5 h. The phage buffer was then collected and filtered through a 0.22 µm pore-size filter.

### Infectivity of phages on multiple strains

The infectivity of the isolated phages was evaluated using a spot assay with four *R. gnavus* strains (ATCC 29149^T^, ATCC 35913, NBRC 114413, and JCM 31371). All phages were adjusted to a concentration of 1 × 10^8^ PFU/mL, and 10-fold serial dilutions (10^0^ to 10^−7^) were prepared in phage buffer. A 0.4% mGAM-CGA soft agar-containing indicator strain was overlaid onto mGAM-CGA agar plates, and 2 μL of each phage suspension was spotted on it. Plates were incubated anaerobically at 37°C for 18–24 h. Plaque formation was assessed visually. The EOP was calculated by defining the titer on ATCC 35913 as 1 and expressed as the mean ± standard deviation (SD) from three independent experiments (*N* = 3). In cases where LO was observed, the lowest dilution at which LO occurred was recorded along with the dilution factor (10^0^–10^7^).

### Killing assay

OD_530_ was measured using a Vi-SPEC II spectrophotometer (Kyokuto, Japan) inside an anaerobic chamber, whereas OD_600_ was measured using a microplate reader (Infinite M Nano, TECAN, Austria) for killing assays. *R. gnavus* ATCC 35913, ATCC 29149^T^, NBRC 114413, and JCM 31371 were cultured anaerobically in mGAM-CGA broth at 37°C until the cultures reached an OD_530_ of 0.3 and were then diluted to 5.0 × 10^7^ CFU/mL. Phages were added at MOIs of 1, 0.1, or 0.01. Microplates were sealed with Titer Stick HC Film (Watson, Japan) and incubated at 37°C. Bacterial growth was monitored by measuring the OD_600_ at 30 min intervals for 24 h using a microplate reader. All experiments were performed in triplicate (*N* = 3). Killing kinetics are presented as raw OD_600_ values over time. The growth inhibition rate (%) was calculated using the OD_600 phage_ and OD_600 control_ values at a specific time point. Both OD_600 phage_ and OD_600 control_ values were defined by subtracting the OD_600_ values of the medium from the raw OD_600_ values. The OD_600 phage_ refers to the OD_600_ measurements when host bacteria were co-cultured with phages. The OD_600 control_ referred to measurements taken without the addition of the phage. The growth inhibition rate was then calculated as follows:

Growth inhibition rate (%) = (1 – OD_600 phage_ / OD_600 control_) × 100 OD_600_ values at 4, 8, 12, 16, and 20 hpi were extracted to calculate the growth inhibition rate (%), as shown in Fig. 1. Values greater than 100% and lower than 0% were set to 100% and 0%, respectively.

### Generation of phage-resistant bacteria and PCR detection of phage-derived sequences

Phage-resistant bacteria were generated by spotting 2 μL of phage lysate (1 × 10^8^ PFU/mL) onto mGAM-CGA agar plates overlaid with 0.4% soft agar containing *R. gnavus* ATCC 35913. Plates were incubated at 37°C until bacterial regrowth was observed in the lysis zone. Regrown bacteria were isolated and streaked three times to obtain single colonies.

Colony PCR was performed to detect phage-derived sequences in phage-resistant bacteria. PCR amplification was carried out using phage-specific primers targeting conserved regions of the phage genome (Table S5). Reactions were performed in a total volume of 10 µL containing 1 µL of template, 1.25 µM of each primer, and 5 µL of Q5 Hot Start High-Fidelity 2× Master Mix (New England Biolabs, USA). PCR cycling conditions were as follows: initial denaturation at 98°C for 30 s, followed by 30 cycles of 98°C for 10 s, 60°C for 30 s, and 72°C for 20 s, and a final extension at 72°C for 2 min. PCR products were analyzed by agarose gel electrophoresis. Colonies of the parental host strain (*R. gnavus* ATCC 35913) were used as negative controls to confirm the absence of phage-derived sequences. Purified phage lysates were used as positive controls. For phages in which the phage-derived sequence could not be confirmed, an additional 20 colonies were examined.

### Preparation of phage genomic DNA for next-generation sequencing

A 5 mL phage lysate with a titer of at least 1 × 10^8^ PFU/mL was treated with 4 μL of TURBO DNase (Invitrogen, USA) and 5 μL of RNase A (Nippon Gene, Japan) at 37°C for 1 h. Phages were precipitated with 0.5 M NaCl and 10% (wt/vol) polyethylene glycol (PEG) #6000 (Nacalai Tesque, Japan). The resulting precipitate was resuspended in 1 mL of phage buffer. This solution was mixed with 5 μL of 0.5 M EDTA (pH 8.0) and 1 μL of 20% SDS (Wako) and incubated for 20 min at 60°C with gentle shaking.

The sample was mixed with 300 µL of phenol-chloroform-isoamyl alcohol (Nacalai Tesque) and centrifuged. The aqueous phase was transferred and mixed with 300 μL of chloroform-isoamyl alcohol (Nacalai Tesque) and centrifuged. DNA was precipitated by adding 100 µL of 3 M sodium acetate (Nacalai Tesque) and 700 µL of isopropanol (Nacalai Tesque), followed by centrifugation. The resulting pellet was washed with 70% ethanol (Nacalai Tesque) and resuspended in 200 μL water. The extracted DNA was further purified using a Genomic DNA Clean and Concentrator-10 Kit (Zymo Research, USA) prior to DNA sequencing.

### Phage genome sequencing and bioinformatics analysis

Whole-genome sequencing was performed using the NovaSeqX, NextSeq system (Illumina, USA). Libraries were prepared using Illumina DNA Prep (M) Tagmentation Kit and sequenced in paired-end mode. Raw reads were quality-filtered using BBDuk in BBMap v38.84 (https://github.com/BioInfoTools/BBMap) and assembled into contigs and scaffolds using SPAdes v4.0.0 (48). Initial annotation was performed using Pharokka v1.7.5 as default settings (49). CDSs were predicted using PHANOTATE (50), tRNAs using tRNAscan-SE 2.0 (51), tmRNAs using Aragorn (52), and CRISPRs using CRT (53). Functional annotation was generated by matching each CDS to the PHROGs (54), VFDB (55), and CARD (56) databases using MMseqs2 (57) and PyHMMER (58). Subsequently, additional functional annotation was conducted using Phold v0.2.0 (https://github.com/gbouras13/phold), also with default settings, to complement the sequence-based annotations with structural homology-based prediction (54, 59–61). All phage genomes were assembled as circular contigs and manually reoriented to start at the terminase small subunit gene for consistency. BLASTn searches were performed using the NCBI BLAST web interface (https://blast.ncbi.nlm.nih.gov) in February 2026. Searches were conducted using the standard BLASTn algorithm with default parameters against the NCBI Core nt database. To assess similarity to known phages, searches were restricted to viral sequences (taxid: 10239). To identify homologous regions within bacterial genomes, additional BLASTn searches were performed against the NCBI Core_nt database with viral sequences excluded (NOT taxid: 10239). Additionally, pairwise nucleotide sequence comparisons were performed using the “Align two or more sequences” function of the NCBI BLAST web interface. Genomes of 29 RgGU phages were used as the query sequence, and complete genome sequences of *R. gnavus* strains (excluding JCM 31371) were used as subjects.

Intergenomic similarity among phage genomes was calculated using VIRIDIC v1.1 (62). Proteomic relationships among the phages were analyzed using ViPTree v4.0 (63). Whole-genome sequences were uploaded to the ViPTree server, and whole-proteome similarity was calculated based on tBLASTx comparisons. The resulting proteomic dendrogram was visualized to determine the proteomic relationship of the isolated phages relative to previously reported *R. gnavus* phages and 2,260 phage genomes. For additional phage classification, the 29 newly isolated phages, together with seven previously reported *R. gnavus* phages (accession numbers: PQ571303 and MT980836–MT980841), were analyzed using VirClust (26). Clustering trees were cut at a distance threshold of 0.625 to define putative subfamily-level groups. Protein-level conservation was assessed using DiGAlign v2.0 through pairwise and multiple alignments (64). Comparative analyses were performed both within individual clusters and among four representative phages selected to reflect inter-cluster diversity. To aid interpretation, genes were color-coded using a predicted function based on the annotation results. Colors represent functional categories as follows: head and packaging, light blue; DNA, RNA, and nucleotide metabolism, yellow; connector, light cyan; tail, light green; lysis, red; integration and excision, orange; moron, auxiliary metabolic gene, and host takeover, pastel violet; transcription regulation, lavender purple; other, gray; and unknown function, light gray.

### Phage morphology observation by TEM

Phage lysates were prepared as previously described in the Materials and Methods section under “Preparation of phage lysates.” For TEM observation, the phage lysates were further purified as follows. Phage lysates were treated with Benzonase (Merck, Germany) for 2 h at 37°C, followed by the addition of 1 M NaCl and 10% (wt/vol) PEG 6000 to precipitate the phages. After overnight incubation at 4°C, the lysates were centrifuged, and the phage pellet was resuspended in phage buffer. The phages were then purified by two rounds of ultracentrifugation in CsCl (Wako); the CsCl was removed using Amicon Ultra Centrifugal Filters, 100,000 MWCO (Merck), according to the manufacturer’s protocol. Then, 2 µL of purified phage lysate was placed on a grid and incubated for 2 min at RT. Unbound phages were washed with Milli-Q water and negatively stained with uranyl acetate. Phages were imaged using a JEM-2100F (JEOL, Japan). The lengths of the head and tail were measured using ImageJ (NIH, USA; version 1.54g). The mean values and standard deviations of at least seven phage particles are shown in Fig. 5A. The percentage of phages with appendages was determined by analyzing 100 phage particles.

### Temperature and pH stability

To evaluate the thermal stability of the phages, phage suspensions were diluted in PBS (−) to 10^7^ PFU/mL and incubated for 2 h at various temperatures: 4, 22, 37, 50, 60, 70, and 80°C. Similarly, pH stability was assessed by incubating the phage suspensions (10^7^ PFU/mL) at RT for 2 h in PBS (−) adjusted to pH values ranging from 1 to 13 using either hydrochloric acid or sodium hydroxide. Following incubation, the phages were serially diluted 10-fold and spotted onto double-layer agar plates containing ATCC 35913 to quantify viable phage particles. Experiments were performed in triplicate (*N* = 3), and the means and SD are presented as graphs.

### Adsorption assay

For assessing the phage adsorption to ATCC 35913, a mixture comprising 1.7 mL of *R. gnavus* culture (OD_530_ = 0.3, 10^8^ CFU/mL) and 170 µL of phage suspension (10^5^ PFU/mL) was prepared and aliquoted into several tubes (400 µL each). All the tubes were incubated at 37°C. At each time point, the tubes were centrifuged, and 300 µL of supernatant was collected. Chloroform was added to the supernatant, mixed, and centrifuged again to recover the supernatant (150 µL). Then, 10–50 µL of the supernatant was plated with the indicator strain (ATCC 35913), and the resulting plaques were counted. For analyzing the adsorption of RgGU1 to ATCC 29149^T^, chloroform was added without centrifugation. The experiments were repeated three times, and the percentage of unadsorbed phages was calculated by setting the number of input phages to 100%. Data are presented as mean ± SD.

### One-step growth curve

*R. gnavus* was sub-cultured until the culture reached an OD_530_ of 0.3, and the phages were inoculated at an MOI of 0.1. Next, 10 min after infection at 37°C, unadsorbed phages were removed by centrifugation, and the cell pellet was washed and resuspended in fresh medium. Infected cells were diluted at least 10^3^-fold and incubated at 37°C without shaking. Aliquots of the culture were collected every 15 min, and the number of phages was counted using the double-layer agar method, with ATCC 35913 as the indicator strain. Burst size was calculated by dividing the mean phage titer at final three time points during the plateau phase by the titer at 30 min, corresponding to the post-adsorption time point. To generate a one-step growth curve, the relative phage titer was calculated by defining the titer at 30 min as 1 PFU/cell.

## Data Availability

FASTQ files generated from NGS were deposited in the DDBJ Sequence Read Archive under accession numbers DRR718858–DRR718886. The 29 phage genomes isolated in this study are available from DDBJ/ENA/GenBank under accession numbers LC889024–LC889052 (Table 2).

## SUPPLEMENTAL MATERIAL

Supplemental material spectrum.03672-25-s0001.pdf Fig. S1 to S4; legends for Tables S1 to S5.

Table S1 spectrum.03672-25-s0002.xlsx Predicted prophage regions identified in *R. gnavus* host genomes.

Table S2 spectrum.03672-25-s0003.xlsx Predicted antiphage defense systems encoded in *R. gnavus* host genomes.

Table S3 spectrum.03672-25-s0004.xlsx BLASTn results of newly isolated *R. gnavus* phages against the NCBI Core_nt viral database.

Table S4 spectrum.03672-25-s0005.xlsx BLASTn identification of bacterial genomic regions homologous to newly isolated *R. gnavus* phages (Core_nt database excluding viral sequences).

Table S5 spectrum.03672-25-s0006.xlsx Primers used for PCR-based detection of phage-derived sequences in phage-resistant colonies of *R. gnavus* ATCC 35913.

ASM does not own the copyrights to Supplemental Material that may be linked to, or accessed through, an article. The authors have granted ASM a non-exclusive, world-wide license to publish the Supplemental Material files. Please contact the corresponding author directly for reuse.
