# Supplementary material for: Extensive library of Ruminococcus gnavus phages revealing genomic and phenotypic diversity with novel clusters
Source: Microbiol Spectr. 2026 Jun 22;14(8):e03672-25. doi: 10.1128/spectrum.03672-25 (PMC13435783; doi:10.1128/spectrum.03672-25)
Supplement: Supplementary file 1 [file spectrum.03672-25-s0001.pdf]

Supplemental Materials

Supplemental Figures

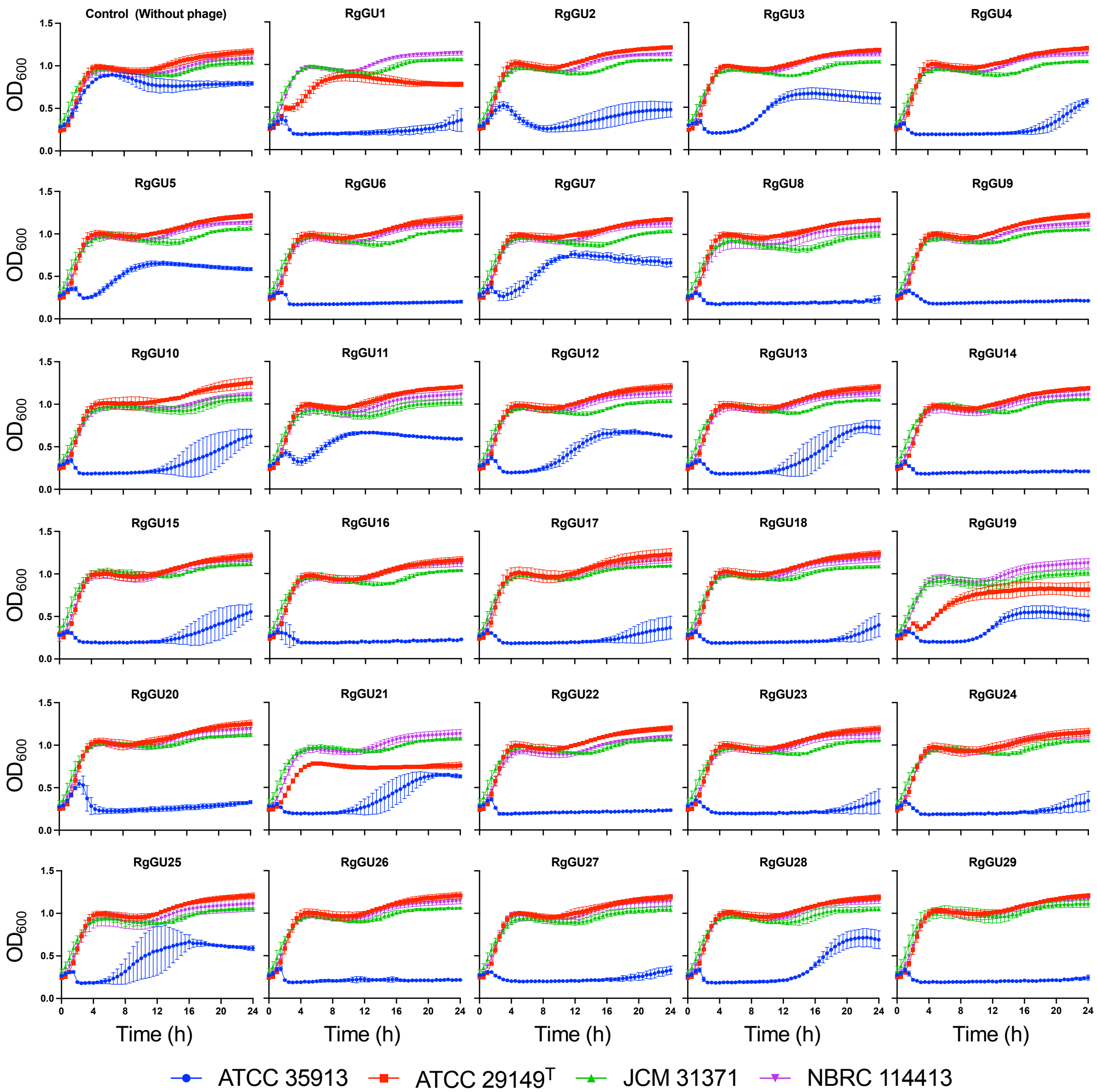

Fig. S1. Killing kinetics assay of 29 *R. gnavus* phages.
Killing curves of *R. gnavus* strains ATCC 35913, ATCC 29149^T, JCM 31371, and NBRC 114413 infected with 29 *R. gnavus* phages at an MOI of 1. Bacterial growth without the phage was used as a control. All experiments were performed in triplicate, and the error bars represent SD.

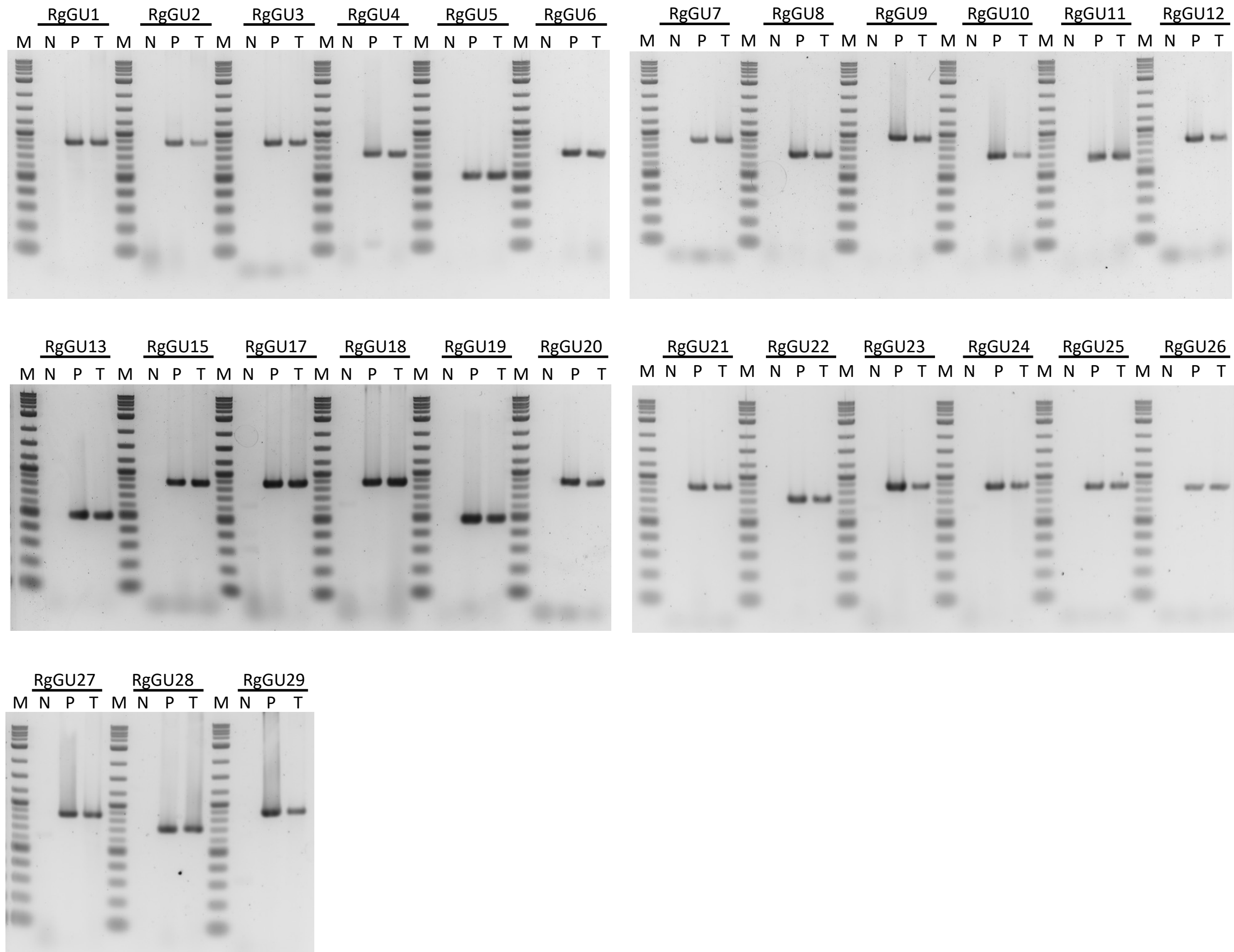

Fig. S2. PCR detection of phage-derived sequences in phage-resistant colonies of *R. gnavus* ATCC 35913.

Colony PCR was performed to detect phage-derived genomic fragments in colonies that regrew within lysis zones following phage exposure. Resistant colonies were subjected to three rounds of single-colony isolation prior to PCR analysis. PCR products were separated by agarose gel electrophoresis. Lane M, 1 kb Plus DNA Ladder; Lane N, colony of parental *R. gnavus* ATCC 35913 (negative control); Lane P, purified phage lysate (positive control); Lane T, representative phage-resistant colony used as PCR template.

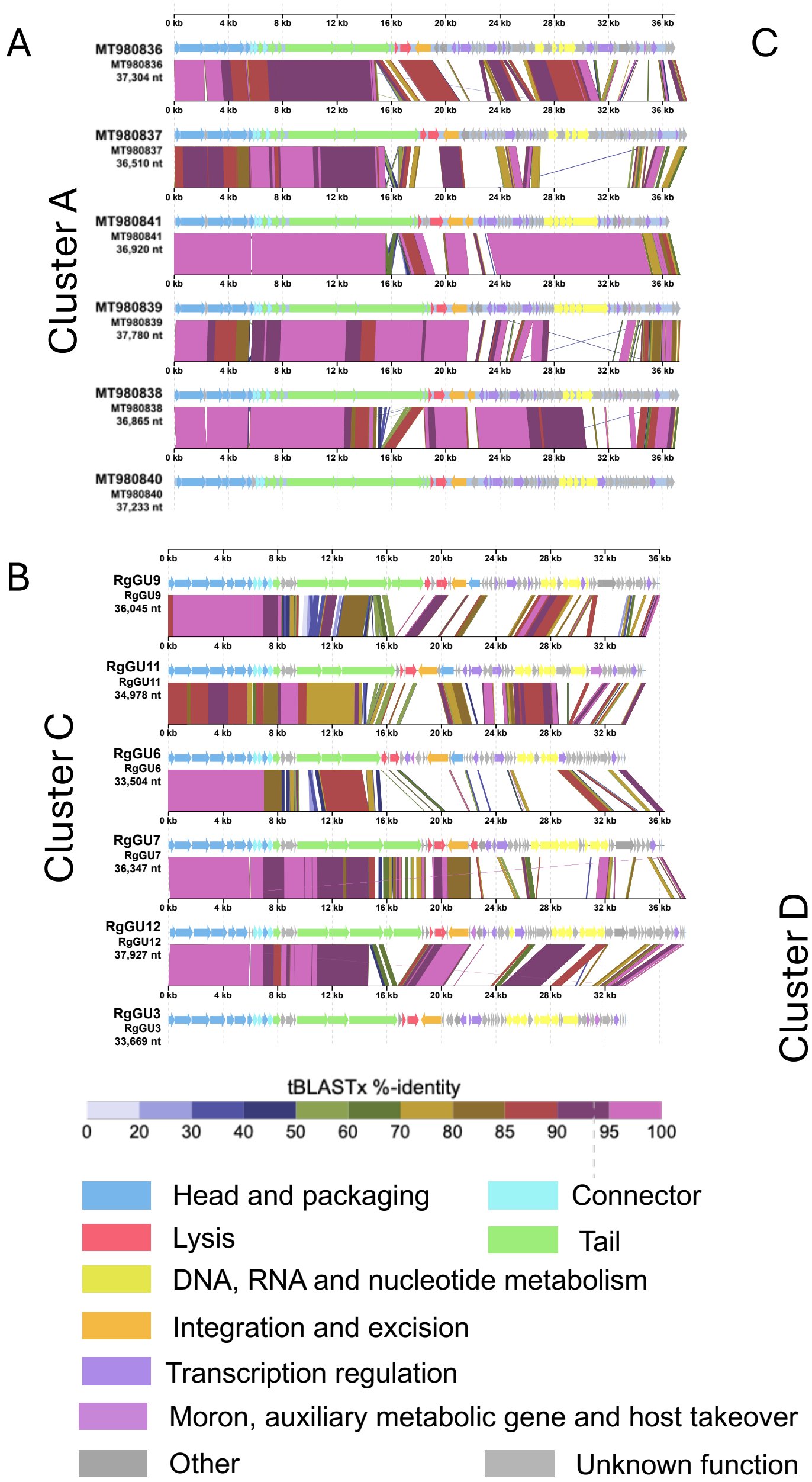

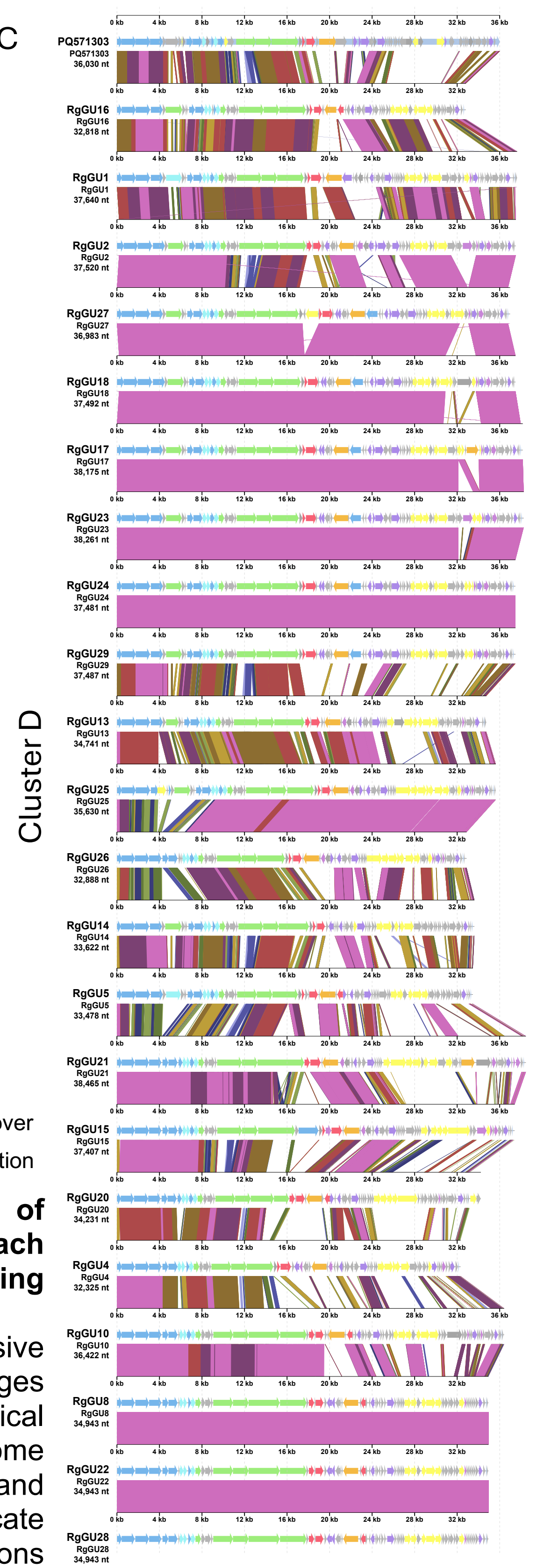

Fig. S3. Protein-level genome alignment of representative *R. gnavus* phages within each VirClust-defined clusters, performed using DiGAlign.

Protein-level genome alignments revealed extensive conservation of gene organization among phages within the same cluster, supporting the hierarchical groupings inferred from the whole-proteome clustering analysis. (A) Cluster A, (B) Cluster C, and (C) Cluster D are shown. Darker regions indicate conserved gene blocks, whereas lighter regions correspond to more variable or unique genomic regions. Cluster labels correspond to those defined by the VirClust-based clustering analysis (Fig. 3B).

A

B

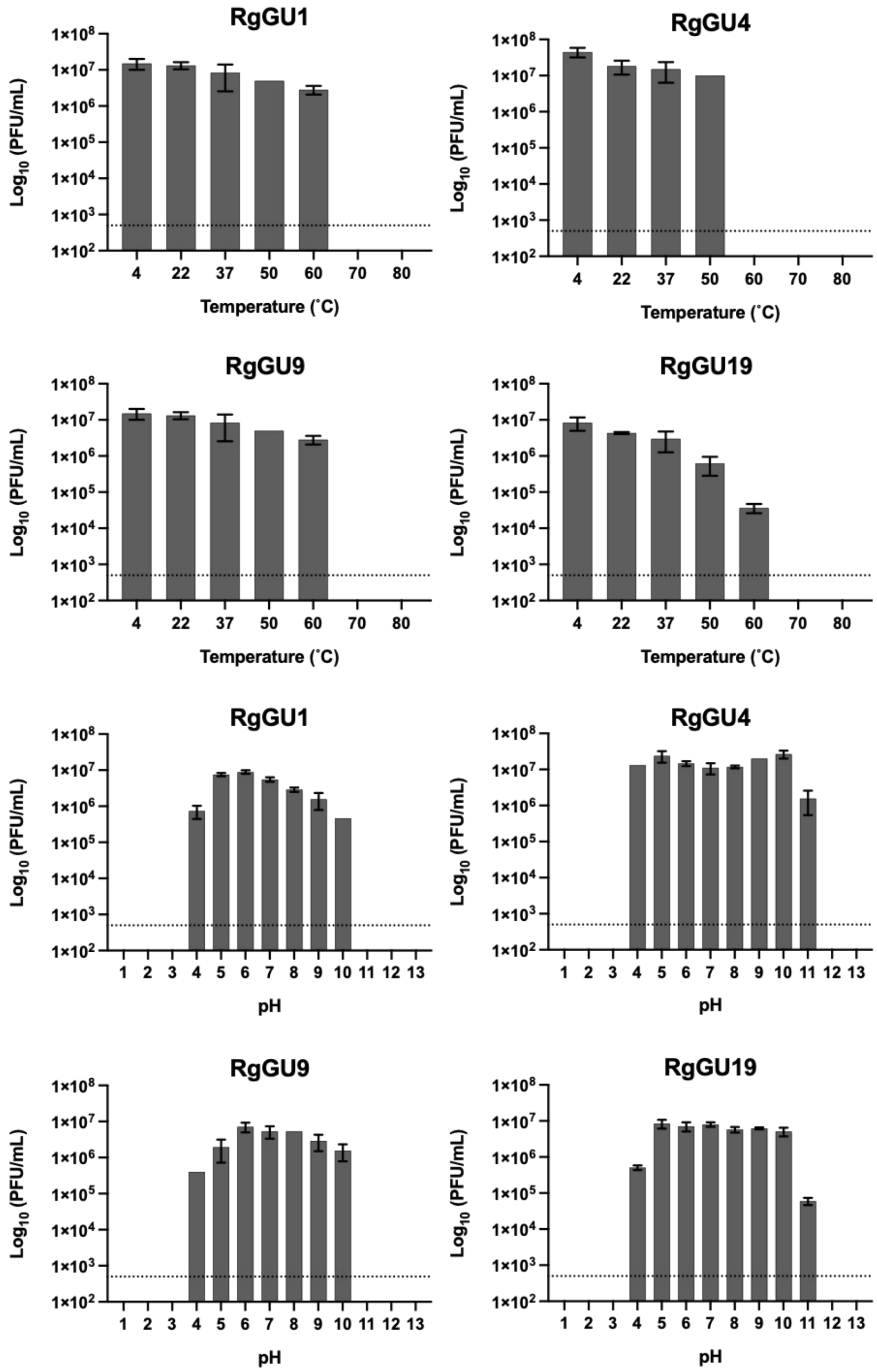

Fig. S4. Stability testing of representative *R. gnavus* phages.
Phages were incubated for 2 h at (A) the temperatures or (B) pH values shown in the graphs. The phage titer after incubation was measured using the double-layer method. Experiments were independently repeated three times, and the mean ± SD is shown in the graphs. The detection limit (shown as dotted lines) was 500 PFU/mL.

Supplemental table legends

Table S1. Predicted prophage regions identified in *R. gnavus* host genomes.
Prophage regions were predicted using PHAge Search Tool Enhanced Release (PHASTER). Results are presented in separate sheets corresponding to each bacterial strain. For each predicted region, the following parameters are reported as provided by PHASTER: Region ID, region length (bp), completeness classification (intact, questionable, or incomplete), PHASTER score, total number of predicted proteins, genomic coordinates (region position), most common related phage, and GC content (%). Regions classified as “intact” represent high-confidence prophage predictions based on PHASTER scoring criteria, whereas “questionable” and “incomplete” regions indicate lower-confidence or fragmented prophage remnants.

Table S2. Predicted antiphage defense systems encoded in *R. gnavus* host genomes.
Putative antiphage defense systems were identified using PADLOC v2.0.0 with default parameters. Results are presented in separate sheets corresponding to each bacterial strain. For each predicted defense-associated gene, the following parameters are reported as provided by PADLOC: genomic start position, genomic end position, strand orientation, predicted defense system name, HMM model name, predicted protein name, and functional description. Each system represents a putative defense locus comprising one or more genes associated with known or predicted antiphage defense mechanisms. System classification and protein annotations were assigned based on hidden Markov model (HMM) matches against the PadlocDB v2.0.0 database.

Table S3. BLASTn results of newly isolated *R. gnavus* phages against the NCBI Core_nt viral database.
BLASTn searches were performed using the NCBI BLAST web interface against the NCBI Core_nt database, restricted to viral sequences (taxid: 10239), in February 2026. Searches were conducted using the standard BLASTn algorithm with default parameters. Results are presented in separate sheets corresponding to each phage (RgGU1–RgGU29). For each genome, the top 100 hits returned by the NCBI web interface are shown. Hits are ranked according to the default BLAST scoring system. The following parameters are reported as provided by the BLAST output: Description, Scientific Name, Max Score, Total Score, Query Cover (%), E value, Percent Identity (%), Accession Length (Acc. Len), and Accession number.

Table S4. BLASTn identification of bacterial genomic regions homologous to newly isolated *R. gnavus* phages (Core_nt database excluding viral sequences).
BLASTn searches were performed using the NCBI BLAST web interface against the NCBI Core_nt database, excluding viral sequences (NOT taxid: 10239), in February 2026. Searches were conducted using the standard BLASTn algorithm with default parameters. Results are presented in separate sheets corresponding to each phage (RgGU1–RgGU29). Hits are ranked according to the default BLAST scoring system. Only hits with query coverage ≥60% and percent identity ≥60% were retained. The reported parameters include Description, Scientific Name, Max Score, Total Score, Query Cover (%), E value, Percent Identity (%), Accession Length (Acc. Len), and

Accession number.

Table S5. Primers used for PCR-based detection of phage-derived sequences in phage-resistant colonies of *R. gnavus* ATCC 35913.
Primers used for PCR amplification of phage-derived genomic fragments in phage-resistant colonies of *R. gnavus* ATCC 35913. For each phage isolate, the forward and reverse primer names and their nucleotide sequences (5′–3′) are listed. Primers were designed to target specific regions within the corresponding phage genomes to detect the presence of phage-derived DNA in bacterial colonies following phage exposure.
